# The contribution of cellulosomal scaffoldins to cellulose hydrolysis by *Clostridium thermocellum* analyzed by using thermotargetrons

**DOI:** 10.1186/1754-6834-7-80

**Published:** 2014-05-29

**Authors:** Wei Hong, Jie Zhang, Yingang Feng, Georg Mohr, Alan M Lambowitz, Gu-Zhen Cui, Ya-Jun Liu, Qiu Cui

**Affiliations:** 1 Shandong Provincial Key Laboratory of Energy Genetics, Qingdao Institute of Bioenergy and Bioprocess Technology, Chinese Academy of Sciences, Qingdao 266101, P R China; 2 Laboratory of Biofuels, Qingdao Institute of Bioenergy and Bioprocess Technology, Chinese Academy of Sciences, Qingdao 266101, P R China; 3 University of Chinese Academy of Sciences, Chinese Academy of Sciences, Beijing 100049, P R China; 4 Departments of Molecular Biosciences and Chemistry, Institute for Cellular and Molecular Biology, University of Texas at Austin, Austin, TX 78712, USA

**Keywords:** Biofuels, Cellulosic ethanol, Cellulosome-cell synergy, Consolidated bioprocessing, Thermophile

## Abstract

**Background:**

*Clostridium thermocellum* is a thermophilic anaerobic bacterium that degrades cellulose by using a highly effective cellulosome, a macromolecular complex consisting of multiple cellulose degrading enzymes organized and attached to the cell surface by non-catalytic scaffoldins. However, due largely to lack of efficient methods for genetic manipulation of *C. thermocellum*, it is still unclear how the different scaffoldins and their functional modules contribute to cellulose hydrolysis.

**Results:**

We constructed *C. thermocellum* mutants with the primary scaffoldin CipA (cellulosome-integrating protein A) truncated at different positions or lacking four different secondary scaffoldins by using a newly developed thermotargetron system, and we analyzed cellulose hydrolysis, cellulosome formation, and cellulose binding of the mutants. A CipA truncation that deletes six type I cohesin modules, which bind cellulolytic enzymes, decreased cellulose hydrolysis rates by 46%, and slightly longer truncations that also delete the carbohydrate binding module decreased rates by 89 to 92%, indicating strong cellulosome-substrate synergy. By contrast, a small CipA truncation that deletes only the C-terminal type II dockerin (XDocII) module detached cellulosomes from the cells, but decreased cellulose hydrolysis rates by only 9%, suggesting a relatively small contribution of cellulosome-cell synergy. Disruptants lacking any of four different secondary scaffoldins (OlpB, 7CohII, Orf2p, or SdbA) showed moderately decreased cellulose hydrolysis rates, suggesting additive contributions. Surprisingly, the CipA-ΔXDocII mutant, which lacks cell-associated polycellulosomes, adheres to cellulose almost as strongly as wild-type cells, revealing an alternate, previously unknown cellulose-binding mechanism.

**Conclusions:**

Our results emphasize the important role of cellulosome-substrate synergy in cellulose degradation, demonstrate a contribution of secondary scaffoldins, and suggest a previously unknown, non-cellulosomal system for binding insoluble cellulose. Our findings provide new insights into cellulosome function and impact genetic engineering of microorganisms to enhance bioconversions of cellulose substrates.

## Background

The cellulosome is a supermolecular machine that is secreted into the growth medium or attached to the cell wall of celluloytic bacteria and consists of multiple structural scaffoldins and enzymatic subunits that interact with each other to efficiently degrade lignocellulose substrates [1,2]. The cellulosome systems of different cellulolytic bacteria have various architectures and components [3]. Among cellulosome-producing bacteria, the thermophilic anaerobic bacterium *Clostridium thermocellum* contains one of the most efficient cellulosome systems for the hydrolysis of lignocellulose substrates. As *C. thermocellum* is also an ethanologen, it is considered one of the most promising candidates for biomass conversion via consolidated bioprocessing and has been extensively studied as a model system for understanding cellulosome structure and function [4,5].

**Figure 1:**
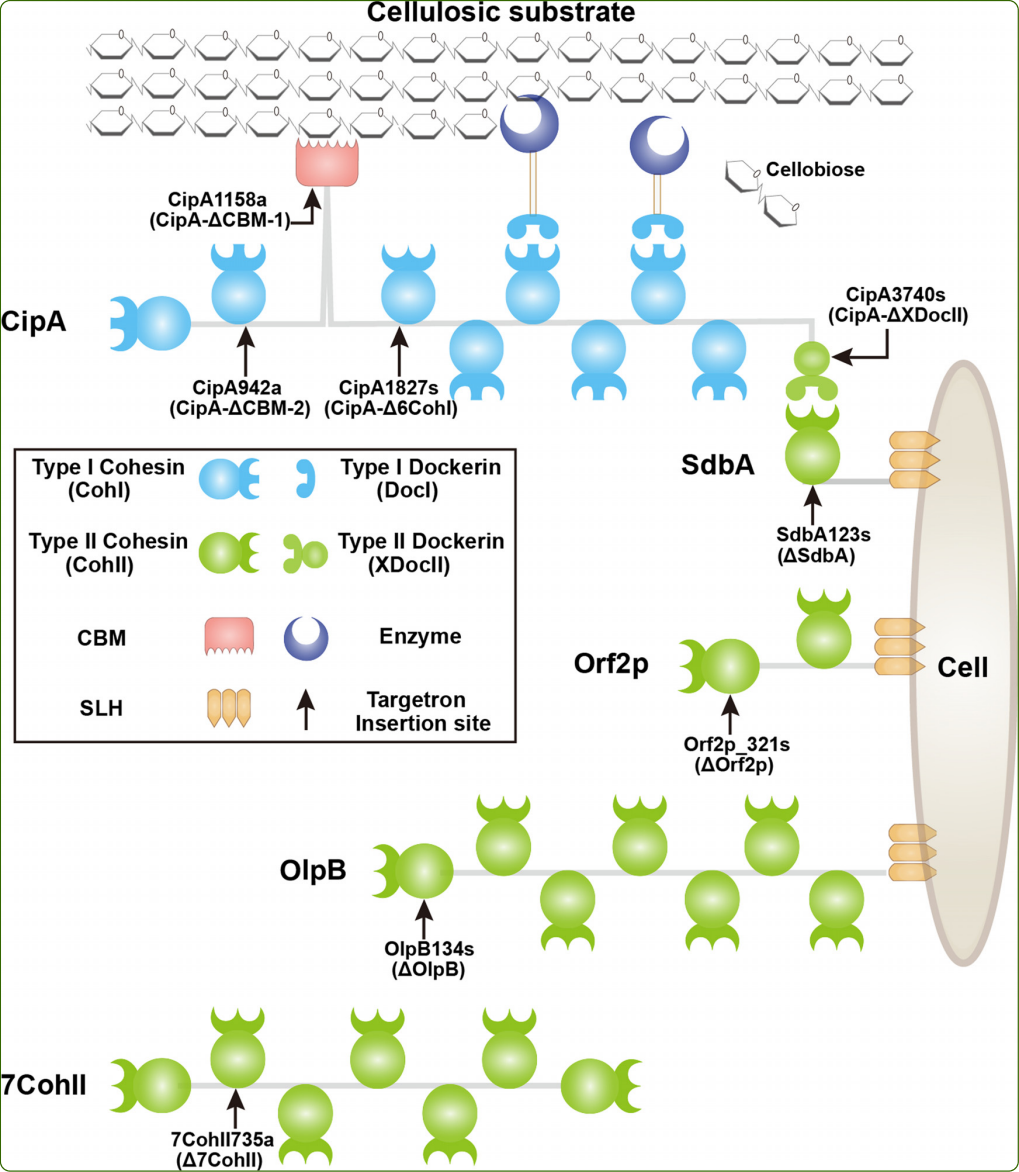
**Schematic representation of the analyzed primary scaffoldin CipA and secondary scaffoldins of *C. thermocellum *DSM1313.** CipA was truncated to delete different modules by inserting targetrons at the positions indicated by vertical arrows to generate the mutants indicated in parentheses. Secondary scaffoldins were disrupted by inserting targetrons at sites near the N-terminus of each open reading frame. Three secondary scaffoldins, SdbA, Orf2p, and OlpB, contain an S-layer homology module that anchors the protein to the cell surface, while 7CohII (Clo1313_1487) is a non-anchored scaffoldin with seven repeated CohII modules. The number of CohI modules in CipA was determined according to genome annotation and PCR analysis (Additional file 1) and differs from the previously published number for this strain [11].

The *C. thermocellum* cellulosome has a molecular weight of about 100 MDa and may consist of more than 70 proteins, including eight putative scaffoldins [6-8]. The major cellulosome scaffoldins analyzed in this study are diagrammed in Figure 1. The most critical structural component of the *C. thermocellum* cellulosome is the primary scaffoldin CipA (cellulosome-integrating protein A), which binds insoluble cellulose and serves as a scaffold for multiple cellulolytic enzymes [9]. CipA also interacts via its C-terminus with secondary scaffoldins, which anchor CipA and its associated cellulolytic enzymes to the cell wall and lead to the formation of more complex polycellulosome structures [10].

To perform its functions, CipA uses various functional modules. First, CipA contains multiple type I cohesin (CohI) modules, which interact with type I dockerin (DocI) modules of different cellulosomal enzymes [12], and the synergistic effects of enzyme proximity to each other and to substrates are hypothesized to enhance cellulose degradation [13]. Second, the carbohydrate-binding module (CBM) of CipA binds to insoluble cellulose substrates and concentrates cellulolytic enzymes in their proximity, contributing further to the efficiency of cellulose degradation [14]. Some of the cellulolytic enzymes bound by CipA also contain CBMs, which further contribute to the binding of cellulosomes to insoluble cellulose. Finally, a type II dockerin module linked to an X-module (XDocII) at the C-terminus of CipA binds to the type II cohesin (CohII) modules of secondary scaffoldins (SdbA, Orf2p, OlpB, and 7CohII), three of which are anchored to the cell surface via an S-layer homology (SLH) module [10]. The interaction of CipA with the three anchoring secondary scaffoldins tethers the *C. thermocellum* cellulosomes to the cell surface, leading to the hypothesis of the “enzyme-microbe synergy” also referred to here as “cellulosome-cell synergy” [15]. Although the roles of the cellulosomal scaffoldins in cellulose hydrolysis have been investigated [16-18], quantitative information about their contribution to cellulose degradation has been scarce, largely because of the lack of tools for the genetic manipulation of thermophilic anaerobes.

**Figure 2:**
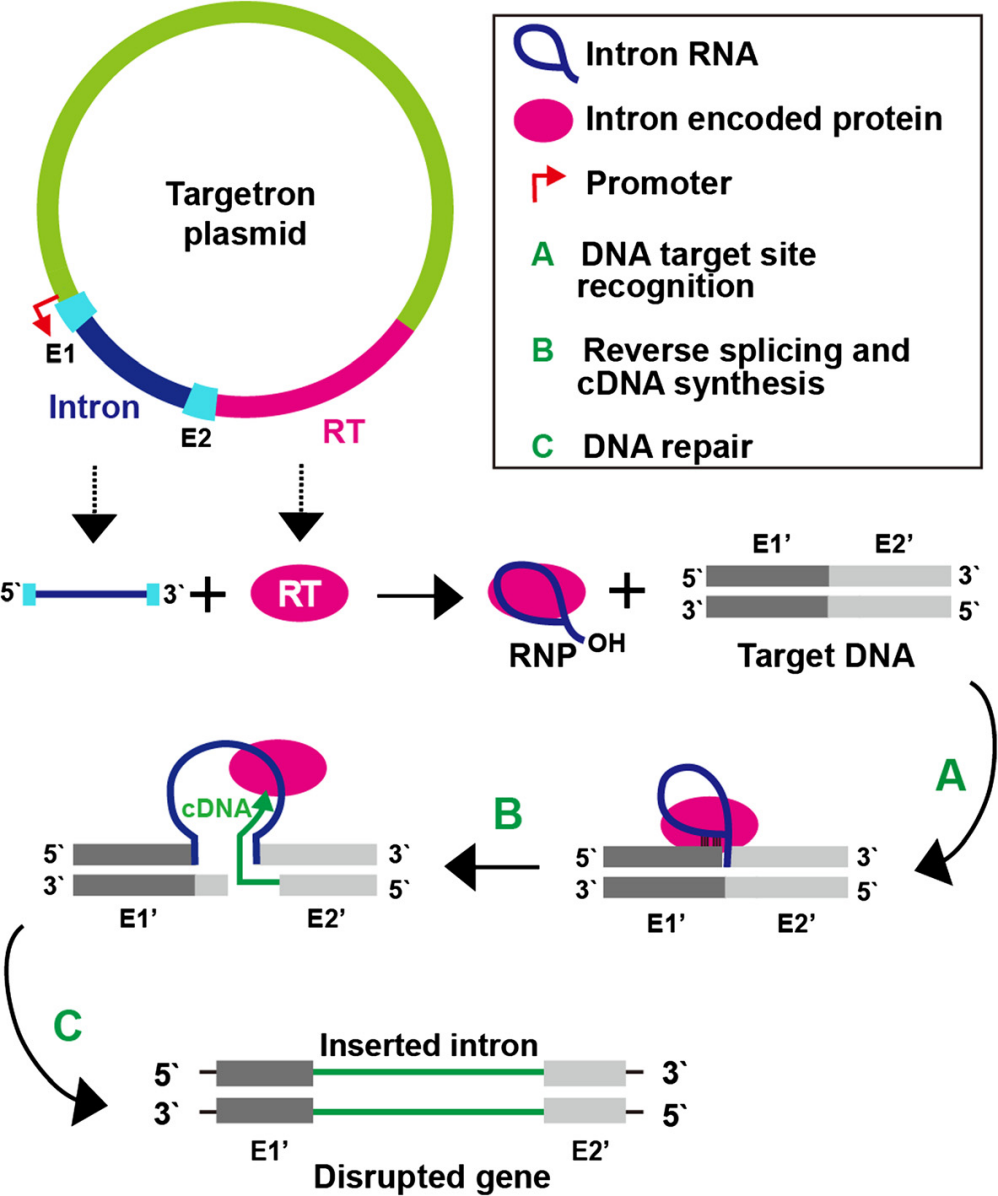
**Thermotargetron method for gene targeting in thermophiles.** A targetron expression plasmid with a strong promoter is used to express a thermostable, approximately 0.8-kb group II intron RNA (TeI3c) and a group II intron reverse transcriptase (TeI4c RT; denoted RT) from the thermophilic cyanobacterium *Thermosynechococcus elongatus*[25,26]. The intron RNA is expressed as a precursor with short flanking exon sequences (denoted E1 and E2) and is spliced from this precursor with the assistance of the intron-encoded RT to yield a ribonucleoprotein (RNP) complex in which the RT is bound to the excised intron lariat RNA. Group II intron RNPs recognize DNA target sites primarily by base pairing of sequence motifs in the intron RNA to the DNA target sequence, with only a small contribution from the intron-encoded RT. The intron RNA then uses its catalytic (ribozyme) activity to insert into the top strand of the DNA target site between target exon sequences (E1’ and E2’) (A), while the DNA endonuclease activity of the RT is used to cleave the bottom strand and the nicked DNA is used as a primer for reverse transcription of the inserted intron RNA (B). The resulting intron cDNA is integrated into the genome by host enzymes (C). Because the DNA target site is recognized mainly by base pairing of the intron RNA, the targetron can be programmed to insert into desired sites simply by modifying the base-pairing motifs in the intron RNA, taking into account the small number of target site nucleotide residues recognized by the RT. The inserted targetron contains multiple stop codons in all three reading frames, resulting in the expression of truncated proteins. Targetron methods are described in detail in [23].

Targetrons are gene targeting tools that are derived from mobile group II introns [19-21]. They use the combined activities of an autocatalytic intron RNA (a “ribozyme”) and an intron-encoded reverse transcriptase (RT) to insert site-specifically into DNA with high efficiency and high and readily programmable DNA target specificity (Figure 2). Targetron insertions disrupt gene expression by introducing multiple stop codons in all three reading frames of the targeted gene. Targetrons are not dependent upon homologous recombination for DNA integration and have been used for genetic engineering of a wide range of bacteria, including a variety of different *Clostridium* species [22-24]. However, all previous targetrons were derived from mesophilic group II introns and do not function efficiently at high temperatures. Recently, we used a mobile group II intron from the thermophilic cyanobacterium *Thermosynechococcus elongatus* to develop a new thermotargetron for gene targeting in thermophiles, and we demonstrated its use in *C. thermocellum*[25].

In this work, we used the thermotargetron method to construct a series of *C. thermocellum* mutants in which the primary scaffoldin CipA was truncated at different positions, or four different secondary scaffoldins (SdbA, Orf2p, OlpB, and 7CohII) were disrupted by targetron insertions. We then used these mutants to analyze the contribution of CipA and its functional modules (CohI, CBM, and XDocII) and secondary scaffoldins and their SLH modules to cellulose degradation. We found that the primary scaffoldin CipA and its CBM are most critical and that all four secondary scaffoldins analyzed are required for maximum rates of cellulose hydrolysis. However, a small C-terminal truncation of CipA that specifically disrupts cellulosome adhesion to the cell had only a minor effect on cellulose hydrolysis rates, suggesting a limited contribution from cellulosome-cell synergy. Surprisingly, the CipA-ΔXDocII mutant adheres to insoluble cellulose almost as strongly as wild-type (WT) cells, despite the absence of cell-associated polycellulosomes. The latter finding suggests that *C. thermocellum* has a previously unknown non-cellulosomal-based system for binding insoluble cellulose.

## Results

### Construction of *C. thermocellum* mutant strains

In *C. thermocellum* DSM1313, the strain used in this study, the major cellulosome scaffoldin CipA harbors eight repeated CohI modules, a CBM, and a C-terminal XDocII module (Figure 1, Additional file 1). The secondary scaffoldins SdbA, Orf2p, OlpB, and 7CohII contain one, two, seven, and seven CohII modules, respectively. The anchoring scaffoldins SdbA, Orf2p, and OlpB also contain a C-terminal SLH module for cellular attachment, whereas the non-anchoring scaffoldin 7CohII contains no SLH module. Previous studies showed that *C. thermocellum* CipA mutants with an IS*1447* insertion in the first CohI module or a complete deletion of the gene via homologous recombination are strongly deficient in cellulose degradation [16,18]. However, targeted mutations in different regions of *cipA* have been difficult to construct by homologous recombination due to the presence of highly repeated DNA sequences within the gene. A CipA mutant lacking the C-terminal XDocII module was constructed by homologous recombination, but required the use of a modified *cipA* allele in which DNA repeats were removed by extensive synonymous substitutions [18]. The latter could affect CipA expression or folding, and detailed analysis of this mutant has not been reported. No secondary scaffoldin mutants had been isolated previously.

Here, we constructed a series of *C. thermocellum* DSM1313 mutants with C-terminal truncations at four different positions in CipA or disruptions in four different secondary scaffoldins (SdbA, Orf2p, OlpB, and 7CohII) by using thermotargetrons designed to insert at specific sites within the target genes encoding those proteins (Figure 2, Additional file 2 and Additional file 3[25]). The CipA mutants are named CipA-ΔXDocII, CipA-Δ6CohI, CipA-ΔCBM-1, and CipA-ΔCBM-2 according to the farthest upstream module that was deleted (Additional file 4). The secondary scaffoldins were disrupted by inserting targetrons close enough to the start codon to delete all the CohII modules in SdbA, Orf2p, and OlpB, and all but one CohII module in 7CohII, yielding the mutants ΔSdbA, ΔOrf2p, ΔOlpB, and Δ7CohII named after the targeted protein (Additional file 2). The precise targetron insertion in each of the mutants was confirmed by polymerase chain reaction (PCR) analysis and sequencing (Additional file 3). Southern hybridizations confirmed single insertions at the expected site for most thermotargetrons, but additional off-target insertions were detected in three mutants (CipA-ΔXDocII, CipA-ΔCBM-2, and ΔSdbA) (see Additional file 3). Thermal asymmetric interlaced (TAIL)-PCR analysis indicated that the off-target insertion in CipA-ΔXDocII is in the gene Clo1313_1971. As discussed below, the CipA-ΔXDocII mutant shows only a small decrease in growth rate or cellulose degradation, and it is thus unlikely that the off-target insertion in this mutant has a substantial effect. Further, quantitative reverse transcription (qRT)-PCR analysis indicated that the thermotargetron insertion in CipA-ΔXDocII did not decrease transcript levels of downstream genes in the *cipA* operon (*olpB*, *orf2p*, and *olpA*[3]) relative to glyceraldehyde 3-phosphate dehydrogenase mRNA used as a standard (Additional file 5). The off-target insertions in CipA-ΔCBM-2 are in Clo1313_2646 (hypothetical protein) and Clo1313_1129 (spore coat protein), and those in ΔSdbA are in Clo1313_1277 (hypothetical protein) and Clo1313_1305 (GH10 xylanase), which is not expressed detectably in cells grown on Avicel, the insoluble microcrystalline cellulose substrate used in this study [27].

**Figure 3:**
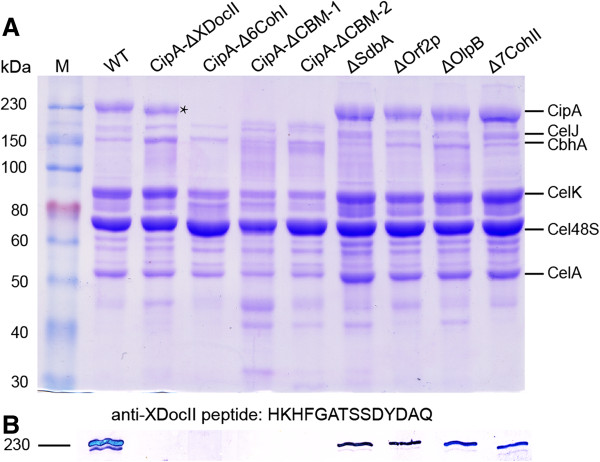
**Analysis of cellulosomal proteins of *C. thermocellum *wild-type and mutant strains.** Cellulosome proteins were isolated by using a modified cellulose-affinity procedure [31] (see Materials and methods) and analyzed by **(A)** sodium dodecyl sulfate (SDS)-polyacrylamide gel electrophoresis (PAGE) with Coomassie blue staining and **(B)** immunoblotting using an antibody directed against a C-terminal XDocII peptide to detect the full-length CipA protein. Six bands corresponding to known cellulosomal proteins are identified to the right of the Coomassie blue-stained gel [9,27]. Intact CipA protein was not detected in any of the CipA-truncated mutants either by staining or immunoblotting, indicating successful gene disruption. Both cellulosomal and extracellular proteins (Additional file 9) of CipA-ΔXDocII contain a band that was slightly smaller than wild-type CipA (indicated by an asterisk) and was identified by mass spectroscopy as a truncated CipA lacking an XDocII module (Additional file 6 and Additional file 7). Secondary scaffoldins are not detectable in Coomassie blue-stained SDS-polyacrylamide gels of purified cellulosomes because they are expressed at low levels, and the affinity purification enriches for proteins that bind directly to cellulose [27,29,30]. M, protein markers.

We analyzed the protein composition of purified cellulosomes prepared by a modified cellulose-affinity procedure [28] by sodium dodecyl sulfate-polyacrylamide gel electrophoresis (SDS-PAGE) and immunoblotting. The results show that full-length CipA is missing in the CipA mutants, but otherwise the composition of major cellulosomal proteins was not changed substantially in any of the mutants (Figure 3). The gels show mainly CipA and associated cellulolytic enzymes. The lane for the CipA-ΔXDocII mutant shows a prominent band that is slightly smaller than WT CipA and was identified by mass spectroscopy as truncated CipA lacking the XDocII module (Additional file 6 and Additional file 7). The four secondary scaffoldins (OlpB, 7CohII, Orf2p, and SdbA) are not detected in cellulosomes prepared by this method, because they are expressed at low levels (OlpB, 8 to 65% of CipA; 7CohII, 8 to 14% of CipA; Orf2p, 8 to 44% of CipA; and SdbA, 5 to 34% of CipA) and the affinity purification enriches for proteins that bind directly to cellulose [27,29,30]. The results show that the composition of the *C. thermocellum* cellulosome is largely unchanged even if some scaffoldin genes are disrupted.

### The functions of CipA modules in cellulose hydrolysis

**Figure 4:**
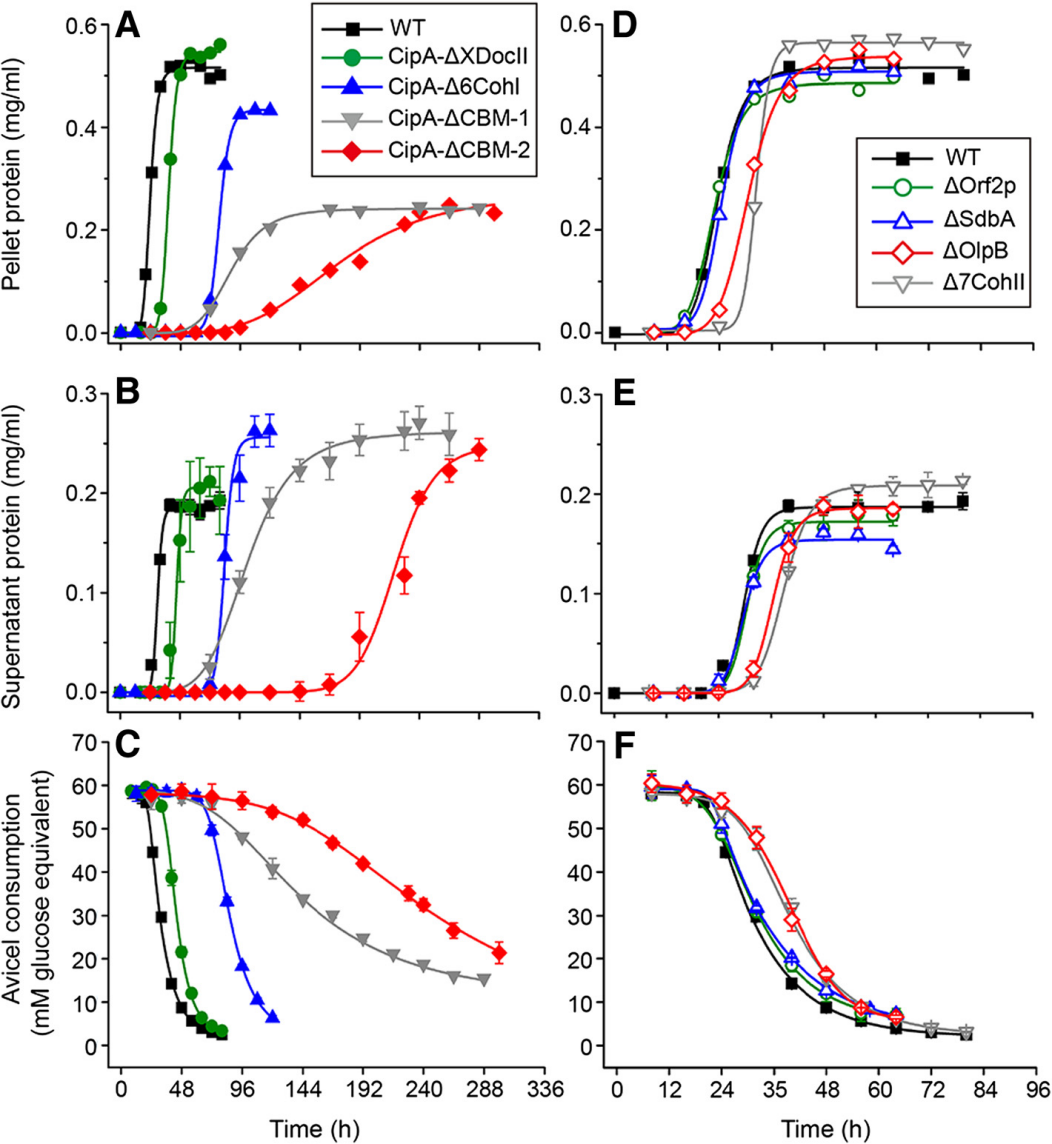
**Growth and fermentation analysis of various *C. thermocellum *mutants with Avicel as carbon source.** Panels **(A, B, C)** show results for CipA-truncation mutants and panels **(D, E, F)** show results for secondary scaffoldin disruption mutants. **(A, D)** cell growth represented by the abundance of total protein in bacterial pellets; **(B, E)** abundance of extracellular protein in the cell culture broth supernatant after pelleting of bacteria; **(C, F)** Avicel consumption in mM glucose equivalents per h. Average values and standard deviations are indicated by error bars and calculated for three replicates for each strain. For measurement of total cell protein, equal amounts of cell culture (0.2 mL) from each of the three replicate experiments were combined before the measurement. The data were fit to a logarithmic growth curve based on a four-parameter equation **(A, B, D, E)** or to a sigmoidal curve based on a five-parameter Richards equation [32]**(C, F)**.

Fermentation experiments were done with the WT and thermotargetron-constructed mutant strains. Either cellobiose or Avicel was used as the sole carbon source. Cellobiose is a degradation product of cellulose and tests the sugar uptake and general sugar metabolism of strains. As expected, the WT and mutant strains grew similarly with cellobiose as the carbon source (Additional file 8). In contrast, the WT and mutants differed dramatically in their ability to grow with Avicel as the carbon source. Generally, all CipA-truncated mutants grew more slowly, produced less total biomass (measured as total protein in equal volumes of pelleted cells), and showed decreased rates of Avicel consumption (Figure 4A and C and see below). These defects became progressively more severe with deletion of more modules from CipA and most severe for the CipA-ΔCBM-1 and CipA-ΔCBM-2 strains, in which the CBM is deleted (Figure 4A). In WT and the CipA-truncated strains, the extracellular proteins showed similar profiles to those of cellulosomal proteins (Figure 3, Additional file 9), but the amount of extracellular proteins was higher in the CipA mutants than that in the WT at the end of fermentation, particularly in those CipA mutants that have larger C-terminal truncations and the strongest inhibition of cellulose hydrolysis (Figure 4B and see below)*.*

**Figure 5:**
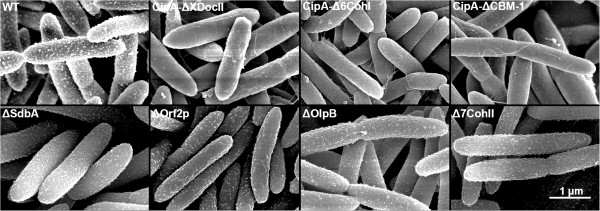
**Scanning electronic microscope visualization of *C. thermocellum *cells using cellobiose as carbon source.** The cell surface of CipA-truncated mutants appears smooth in comparison with the wild type and suggests the loss of polycellulosome structures. No major changes are observed in the appearance or density of polycellulosomes on the cell surface of secondary scaffoldin mutants (Additional file 11). A scale bar is shown at the bottom right.

We examined the effect of the CipA truncations on the adherence of cellulosomes to the cell by scanning electron microscopy (SEM). Figure 5 shows that deletion of the XDocII module in the CipA-ΔXDocII strain causes a dramatic change in the appearance of the cell surface. The CipA-ΔXDocII mutant bacteria have a smooth appearance, whereas the WT cells appear rough. Similarly, bacteria with two longer CipA truncations that also delete the XDocII module (CipA-Δ6CohI and CipA-ΔCBM-1) are also smooth. This suggests that the granules observed on the WT cell surface are cellulosomes [1] and that CipA-ΔXDocII carries few if any cellulosomes on its surface. Thus, our results indicate that the C-terminal XDocII module of CipA is crucial for tethering cellulosomes to the cell surface.

The quantitative influence of the different scaffoldin modules of CipA to cellulose consumption was deduced from fermentation assays of the mutants by determining the maximum hydrolysis rate (*V*_*max*_) and *t*_*max*_, which is the cultivation time at *V*_*max*_[17]. Representative data are shown in Figure 4C, Table 1, and Additional file 10. Although the proposed “cellulosome-cell synergy” [15] should be strongly affected by deletion of the XDocII module of CipA, which abolishes the binding of cellulosomes to the cell wall, we observed only a small effect on cellulose use in the CipA-ΔXDocII mutant (9% decrease of *V*_*max*_ and 1.58-fold longer *t*_*max*_, Table 1). A larger truncation that also deleted six CohI modules from CipA in CipA-Δ6CohI decreased *V*_*max*_ by 46% and increased *t*_*max*_ more than threefold (Table 1, Figure 4C). This decrease may reflect both the decreased number of CohI modules and the decreased levels of this truncated protein, which is not detected by Coomassie blue staining (Figure 3). Slightly longer truncations that also delete the CBM of CipA (CipA-ΔCBM-1 and CipA-ΔCBM-2) decreased *V*_*max*_ by 89 to 92% and increased *t*_*max*_ four to eightfold (89 to 180 h) (see Table 1 and Additional file 10). Thus, without the “cellulosome-substrate synergy” mediated by the CohI modules and the CBMs, the efficiency of cellulose degradation is dramatically reduced. Together, these results indicate that the synergy effect mediated by the CohI modules and the CBMs (“cellulosome-substrate synergy”) is much more pronounced than the synergy effect mediated by the C-terminal XDocII module (“cellulosome-cell synergy”).

**Table 1:** **Cellulose hydrolysis activities of *C. thermocellum *** **strains**

***C. thermocellum *** **strains**	***V***_***max***_**(mM glucose equivalent per h/%)**	***t***_***max ***_**(h/fold)**
WT (DSM1313)^*^	2.84/100	26/1.00
CipA-ΔXDocII	2.58/91	41/1.58
CipA-Δ6CohI	1.54/54	81/3.16
CipA-ΔCBM-1^†^	0.31/11	115/4.42
CipA-ΔCBM-2^†^	0.22/8	206/7.92
ΔSdbA	2.44/86	26/1.00
ΔOrf2p	2.31/81	28/1.08
ΔOlpB	2.19/77	40/1.54
Δ7CohII	2.14/75	36/1.38

^*^V_*max*_ and *t*_*max*_ of the WT strain are set as 100% and one fold, respectively. The curve-fitting parameters are listed in Additional file 10.

^†^The targetron in CipA-ΔCBM-2 inserts at the very end of the second CohI module of CipA, possibly affecting the function of this module and resulting in lower *V*_*max*_ and longer *t*_*max*_ values compared with CipA-ΔCBM-1.

### The contribution of secondary scaffoldins to cellulose hydrolysis

We carried out similar experiments for the four mutants (ΔSdbA*,* ΔOrf2p*,* ΔOlpB, and Δ7CohII) in which different secondary scaffoldins were disrupted. Generally, the inactivation of any single secondary scaffoldin influenced cell growth and the abundance of extracellular proteins less than the longer CipA truncations (Figure 4D and E). The deletion of SdbA, Orf2p, and OlpB, containing one, two, and seven CohII modules, respectively, decreased *V*_*max*_ to 86, 81, and 77% of WT, respectively (Figure 4F, Table 1). The disruption of Orf2p and OlpB also increased *t*_*max*_ by 1.08- and 1.54-fold, respectively (Figure 4F, Table 1), indicating that the disruption of some secondary scaffoldins may delay cellulose hydrolysis. Unlike SdbA, Orf2p, and OlpB, 7CohII does not contain an SLH module but carries seven CohII modules similar to OlpB (Figure 1). Disruption of 7CohII reduced *V*_*max*_ by 25% and increased *t*_*max*_ by 1.38-fold. These values are similar to those of OlpB (23% and 1.54-fold), suggesting that the number of CohI modules is more important than whether or not the scaffoldin is anchored. Together, these findings indicate that disruption of any one secondary scaffoldin cannot be fully compensated by the other secondary scaffoldins, suggesting either non-redundant functions or that the total number of CohII modules is important. In most cases, secondary scaffoldins with more CohII modules contribute more to cellulose hydrolysis than those with fewer CohII modules, presumably by enabling the binding of greater numbers of cellulolytic enzymes. No large change was observed by SEM on the ultrastructure or density of polycellulosome structures on the cell surface of any of the secondary scaffoldin mutants (Figure 5, Additional file 11).

### Mechanism of cell adhesion to cellulose

**Figure 6:**
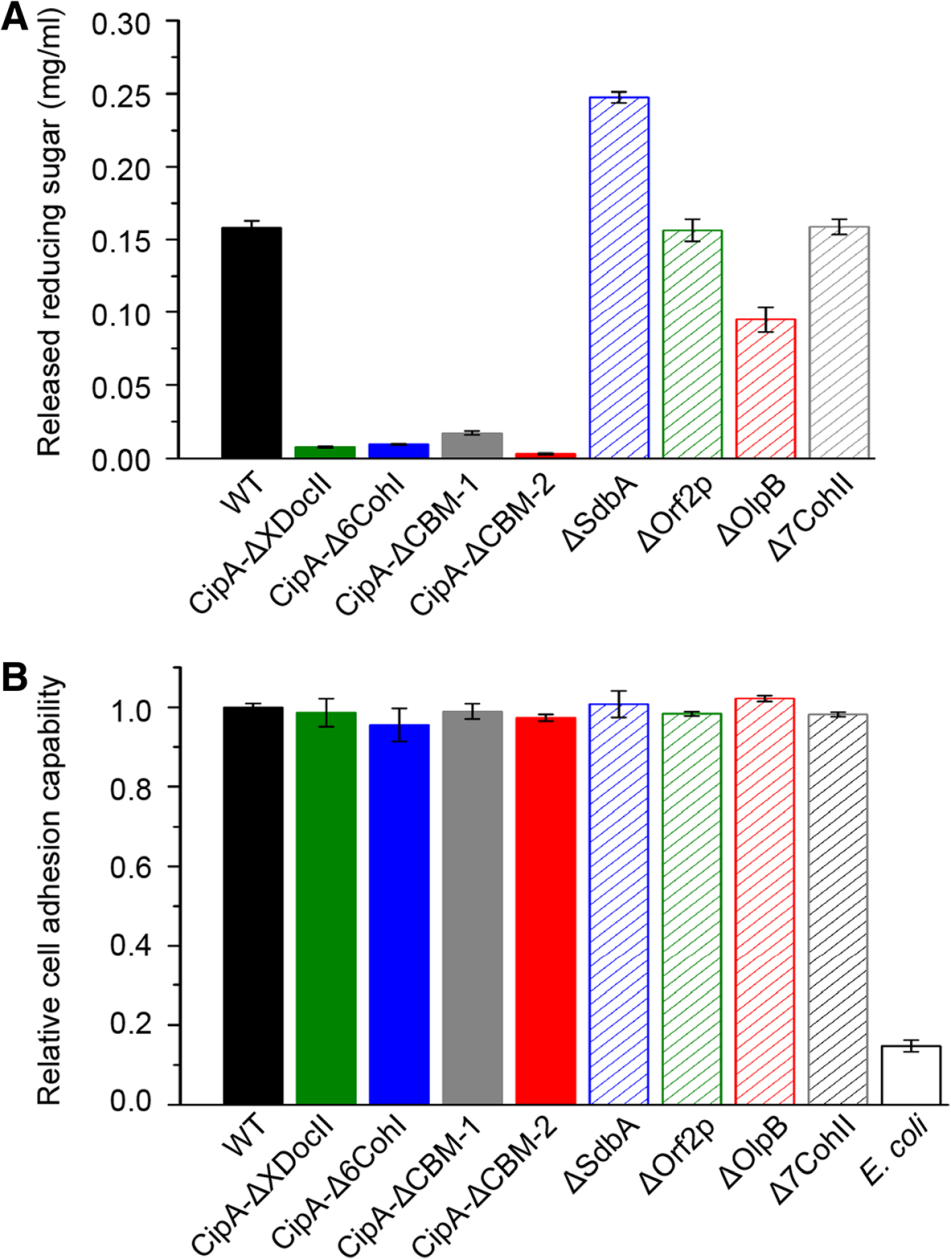
**Cell-associated hydrolysis activity and cellulose-binding ability of wild-type and mutant *C. themocellum *strains. (A)** Cell-associated hydrolysis activity. The bar graphs show the amount of reduced sugars (mg/mL) after hydrolysis of 5 mg Avicel at 55°C for 24 h under oxic conditions measured using the 3,5-dinitrosalicylic acid method [35]. Wild-type cells incubated under the same growth conditions in the absence of Avicel were used as a control to determine the amount of reduced sugar in the absence of cellulose hydrolysis, and this amount was subtracted from the values for the experimental samples. **(B)** Cell adhesion to cellulose. Values for each strain were determined by calculating the percentage of cells attached to a cellulose filter paper (Watermann, 3 × 6.4 cm, 0.34 mm thickness). The values for the mutants are normalized to the percentage of *C. thermocellum* wild-type cells attached to the filter paper (87%), which was set equal to 1. *E. coli* DH5 alpha cells were used as a negative control. Average values and standard deviations indicated by error bars were calculated from three independent experiments.

Cell-associated cellulose hydrolysis and binding assays were performed using cell pellets of various *C. thermocellum* strains under oxic conditions, so that only enzymes already bound to the cell surface could participate in cellulose hydrolysis. These assays were done using *C. thermocellum* cells in the early exponential phase, when WT cellulosomes are located on the cell surface [33,34]. Cells of CipA-disrupted strains had less than 10% hydrolysis activity (measured as released reducing sugar) compared to WT (Figure 6A), which was expected based on the SEM results showing that polycellulosomes are not attached to the cell surface of these mutants. However, the mutants still had >96% of cellulose-binding capability compared to WT (Figure 6B). Thus, surprisingly, the loss of the major cellulosome structure does not substantially influence cellular adhesion to cellulose.The secondary scaffoldin mutants, which retain polycellulosomes on the cell surface under oxic conditions, also had >97% of the cellulose-binding capability of WT cells (Figure 6B). However, the cell associated cellulose hydrolysis activity of ΔOlpB was decreased by 40% compared to the WT (Figure 6A), which might be caused by a change to the internal structure of polycellulosomes bound to the cell surface. Unexpectedly, for unknown reasons, cells of ΔSdbA released 57% more reducing sugar than the WT.

## Discussion

In this study, we used a series of mutants constructed with thermotargetrons to investigate the contributions of primary scaffoldin CipA and four secondary scaffoldins and their functional modules to cellulose hydrolysis by *C. thermocellum*. Our results indicate that the “cellulosome-substrate synergy” mediated by the CBM and CohI modules of CipA plays a major role in cellulose degradation and that each of the four secondary scaffoldins analyzed also contributes. In contrast, we find that the “cellulosome-cell synergy” mediated by the XDocII module of CipA contributes minimally to cellulose degradation by *C. thermocellum* under fermentation conditions. Finally, we find that the adhesion of *C. thermocellum* to insoluble cellulose substrates does not require polycellulosomes attached to the cell surface, as had been widely assumed, but can also occur by a previously unsuspected non-cellulosome-based mechanism.

The primary scaffoldin CipA contains multiple CohI modules, which bind various cellulolytic enzymes and together form the primary cellulosome, as well as a CBM, which targets the cellulosome to insoluble cellulose substrates, and a C-terminal XDocII module, which leads to the attachment of cellulosome to the cell wall [2]. The resulting high local concentration of cellulolytic enzymes in a complex on the cellulose substrate has been suggested to lead to cellulosome-substrate synergy in cellulose degradation compared with free enzymes, but the magnitude of this effect had not been analyzed quantitatively [13,36,37]. The functional modules of CipA are separated by relatively long linker segments, which are rich in proline and threonine residues [38]. The intermodule linkers are intrinsically disordered and flexible, and structural analyses have demonstrated the independent folding of functional modules of CipA, including CohI, CBM, and XDocII [39]. Thus, the deletion of individual modules may not substantially affect the structure or function of the remaining modules. Here, we find that the deletion of six CohI modules of CipA decreased cellulose hydrolysis rates by 46% and the deletion of six CohI modules as well as the CBM decreased rates by 89 to 92%. The effect of deleting only the six CohI modules on cellulose degradation may be overestimated because a decreased concentration of the CipA-Δ6CohI truncated protein would also decrease the number of CBM-binding modules. Notably, deletion of the six CohI modules did not substantially affect the profile of cellulolytic enzymes detected by SDS-PAGE in purified cellulosomes, either because the remaining two CohI modules in CipA provide sufficient binding capacity for these enzymes or because other proteins containing CohI modules (such as OlpA, OlpC) [40] can compensate for those missing from CipA (Figure 3). Thus, we conclude that the CBM, which functions in binding the cellulosome to insoluble cellulose substrates, makes the largest contribution to the efficiency of cellulose degradation by *C. thermocellum*.

In addition to cellulosome-substrate synergy, it has been proposed that the tethering of cellulosomes to the cell surface also enhances cellulose hydrolysis by an “enzyme-microbe synergy” (referred to here as “cellulosome-cell synergy”). Cellulose attachment to the cell could facilitate the removal of inhibitory hydrolysis products, perhaps by disrupting a highly structured water layer at the surface of cellulose, and could also enhance substrate access at the cell surface [15]. Leibovitz and Beguin suggested that binding of cellulosomes to the cell surface is mediated by the type II interaction between the C-terminal XDocII module of CipA and the CohII modules of anchoring scaffoldins (SdbA, OlpB, and Orf2p), which are tightly attached to the cell wall [41]. An X-ray crystal structure has been determined for a high affinity XDocII-CohII complex [42], but a fluorescence assay for docking of purified XDocII modules to the cell surface showed no difference between WT and a CipA deletion mutant, which is expected to have a higher number of unoccupied CohII modules [18]. Here, we show that a small C-terminal truncation of CipA that deletes only the XDocII module completely eliminates cellulosome adhesion to the cells, but surprisingly, the XDocII deletion inhibits cellulose hydrolysis by only 9%. This finding indicates that cellulosome-cell synergy makes only a limited contribution to cellulose degradation under fermentation conditions with microcrystalline cellulose (Avicel) as the carbon source. It remains possible, if not likely, that cellulosome-cell synergy plays a greater role under environmental conditions where the proximity of the cellulosome would increase the local concentrations of cellobiose and glucose, thereby accelerating their uptake by the cell [43].

We find that disruption of each of the four secondary scaffoldins (SdbA, Orf2p, OlpB, or 7CohII) moderately inhibits the rate of cellulose hydrolysis (14 to 25%), indicating either non-redundant contributions or that the total number of CohII modules is important. These four secondary scaffoldins were identified by proteomic analysis [27], but their contributions to cellulose hydrolysis had not been demonstrated previously. We find that disruption of secondary scaffoldins with more CohII modules inhibits cellulose degradation more strongly than disruption of those with fewer CohII modules, irrespective of the presence or absence of an anchoring SLH module. Further, none of the secondary scaffoldin mutants shows a significant decrease in the concentration of cellulosomes on the cell surface. The latter finding could reflect either that the concentration of the remaining secondary scaffoldins is sufficient for cellulosome tethering or that loss of any one secondary scaffoldin can be compensated for by overproduction of other secondary scaffoldins. The mutants we constructed will be useful for investigating whether the different secondary scaffoldins have specialized roles in binding enzymes needed to degrade substrates other than Avicel.

In both WT and mutant strains, the profiles of extracellular proteins secreted into the culture medium are similar to those of purified cellulosomes [29] (Figure 3 and Additional file 9). All of the CipA-truncation mutants have higher concentrations of extracellular proteins in the culture supernatant, resulting from loss of the C-terminal XDocII molecule, which anchors cellulosomes to the cell surface. However, the concentration of extracellular protein increases for CipA mutants with longer truncations and lower cellulose hydrolysis rates. This finding could reflect that decreased levels of cellulose degradation products or other metabolites and/or the lack of intact CipA will trigger a feedback mechanism that leads to the overproduction of extracellular proteins*.* The strategy of using quantity to balance quality would waste resources and energy, leading to the observed decreases in cell growth.

Cellulosomes were described initially as an extracellular cellulose-binding factor [9], and mutants of different *C. thermocellum* strains that are defective in binding cellulose were found to have mutations affecting CipA [16,44,45]. Later, it was shown that cellulosomes of *C. thermocellum* could simultaneously tether to the cell surface and bind cellulose [2]. Thus, it has been widely assumed that the cell binding to insoluble cellulose is mainly mediated by the cellulosome of *C. thermocellum*. We find, however, that the loss of the cellulosomal structures on the cell surface of *C. thermocellum* DSM1313 does not significantly affect binding to insoluble cellulose, which must instead be mediated by other cellular components. The scaffoldins OlpA and OlpC contain both CohI and SLH modules [46], and they could thus contribute to cell adherence to the substrate by binding cellulolytic enzymes carrying DocI modules and CBMs. Other proteins that contribute to cell adherence to cellulose include a hypothetical protein (encoded by Clo1313_0090) of unknown function, which is also predicted to harbor both CBM and SLH domains in *C. thermocellum* DSM1313, and certain anti-sigma factors, which contain both a transmembrane domain and an extracellular CBM [8,47]. Our results are consistent with findings for *C. cellulolyticum*, another cellulosome-producing microorganism, whose cellulose-binding activity is not affected by the deletion of its sole scaffoldin protein CipC [48]. The finding that *C. thermocellum* can efficiently bind insoluble cellulose in the absence of the cellulosome may at least in part explain the relatively small contribution of “cellulosome-cell synergy” to cellulose degradation in our experiments. The previous failure to observe this additional cell-based cellulose-binding mechanism in *C. thermocellum* could reflect differences in the genetic backgrounds of strains used for mutational analysis, secondary mutations in genes other than CipA that affect cellulose binding in the *C. thermocellum* mutants isolated previously, or differences in culture conditions [8]. In nature, both the cellulosome- and non-cellulosome-based mechanisms could contribute to cellulose adhesion by *C. thermocellum* to varying degrees depending on environmental conditions.

Finally, our results demonstrate the utility of targetrons for genetic manipulation in *C. thermocellum*. Here, we used the recently developed thermotargetron system to construct and analyze eight different targeted mutations in *cipA* and four secondary scaffoldin genes, and previously, we used this system to disrupt five additional genes (*hfat*, *hyd*, *ldh, pta*, and *pyrF*; [25]). In comparison, a total of only seven mutants constructed by homologous recombination methods have been reported, two for *cipA* and five for other genes (*ldh*, *pta*, *hpt*, *pyrF*, and *cel48S*) [4,18,49,50]. In addition to targeted gene disruption, targetrons have been used in other bacteria, to insert cargo genes at desired chromosomal locations [51] and for highly efficient large-scale genome engineering [52]. For the latter, targetrons are used to position recombinase (Cre-*Lox*) sites at specified chromosomal sites, where they are acted upon by the recombinase to enable large insertions and multiple sequental or simultaneous deletions without using genetic markers. The polycellulosome system of *C. thermocellum* has evolved over many years to use a combination of degradative enzymes organized by scaffoldins to efficiently degrade cellululose and more complex lignocellulose substrates with greater efficiency than free enzymes [53]. In the future, we anticipate that the use of targetrons will not only continue to increase our understanding of the functions of cellulolytic enzymes and their structural organization by scaffoldins, but will also enable facile engineering of cellulosomes for more efficient production of biofuels and more complex bioconversions of celluloytic biomass than have been found in nature.

## Conclusion

The highly efficient cellulosome of *Clostridium thermocellum* contains both cellulose degrading enzymes and structural scaffoldins. In this study, we constructed a series of mutants to investigate and quantify the contributions of cellulosomal scaffoldins and their interactions to cellulose hydrolysis by *C. thermocellum*. We find that the primary scaffoldin CipA and its carbohydrate-binding module are most critical for efficient cellulose hydrolysis and that secondary scaffoldins also contribute significantly. However, a small C-terminal truncation of CipA that specifically disrupts cellulosome attachment to the cell had only a minor effect on cellulose hydrolysis or binding, suggesting a limited contribution from cellulosome-cell synergy. Our findings provide new insights into cellulosome function and will aid genetic engineering of microorganisms for bioconversions of cellulosic substrates.

## Materials and methods

### Bacterial strains and cultivation

The bacterial strains used in this study are listed in Additional file 2. *Escherichia coli* strains were grown in Luria-Bertani medium with shaking (200 rpm) at 37°C. *C. thermocellum* strains were cultured at 55°C anaerobically in modified GS-2 medium (KH_2_PO_4_ 1.5 g, K_2_HPO_4_ · 3H_2_O 2.1 g, urea 2.1 g, MgCl_2_ · 6H_2_O 1.0 g, CaCl_2_ · 2H_2_O 150 mg, FeSO_4_ · 6H_2_O 1.25 mg, cysteine-HCl 1 g, MOPS-Na 10 g, yeast extract 6.0 g, trisodium citrate · 2H_2_O 3.0 g, resazurin 0.1 mg/L, pH 7.4) [25,54]. Cellobiose (5 g/L) or Avicel (10 g/L) was used as the sole carbon source. Antibiotics were added to the medium at the following concentrations when required: ampicillin, 100 μg/mL; chloramphenicol, 35 μg/mL; thiamphenicol 3 to 6 μg/mL.

### Targetron construction

Targetrons were designed and constructed as described [25]. Briefly, a 357-bp targetron fragment was obtained by two PCRs of pHK-TT1A with overlapping primers (see Additional file 2 and Additional file 3), which change the EBS1, EBS2, IBS1, and IBS2 sequences to target the desired site and introduce SpeI and BsiWI sites for cloning [25]. The PCR fragments were digested with BsiWI and SpeI, and then ligated between the corresponding sites of pHK-TT1A to replace the original targetron fragment [25]. The resulting targetrons are denoted by a number that corresponds to the nucleotide residue 5′ to the targetron insertion site within the target gene, followed by “a” or “s” indicating the antisense/bottom or sense/top strands, respectively (Additional file 2).

### Electroporation and mutant screening of *C. thermocellum*

Targetron plasmids were transformed into *E. coli* BL21(DE3), a *dam*^+^*dcm*^-^ strain, to remove Dcm methylation, thereby improving the transformation efficiency of *C. thermocellum*[55]. The transformation of *C. thermocellum* was done via electroporation as described [25]. Transformants were selected on solid GS-2 medium supplemented with thiamphenicol. Colony PCR of transformants was performed with forward and reverse primers flanking the intron insertion site of the target gene. Targetron insertion gave PCR products that are 0.8 kb larger than that of the WT gene and were sequenced to confirm correct targetron insertion (Additional file 3).

After gene targeting, targetron plasmids were cured by continuous inoculation and growing of cells in fresh medium without antibiotic, and cured strains were verified to be unable to grow in the presence of antibiotic [25].

### Southern hybridization analysis

Southern hybridizations were performed to check the targetron insertion in chromosomal DNA of *C. thermocellum* mutants. Briefly, genomic DNA was isolated from WT and mutant strains using a commercial kit according to the manufacturer’s instructions (Mini-bacterial DNA isolation kit, Tiangen Biotech). DNA was digested with BamHI and EcoRI at 37°C overnight, and then run in a 0.8% agarose gel, which was blotted onto a Nylon membrane (Hybond-NX, GE Healthcare) and hybridized with a DIG-labeled intron probe (DIG-High Prime DNA Labeling and Detection Starter Kit I, Roche), according to the manufacturer’s protocol. The intron probe was generated by PCR with primers Probe172-F and Probe172-R, as described [25]. For those mutants with off-target integrations (more than one band hybridizing to the intron probe), thermal asymmetric interlaced PCR (TAIL-PCR) was performed to identify the additional targetron insertion sites (Genome Walking Kit, Takara, CA, USA). Additional file 2 lists the primers used for TAIL-PCR.

### Quantitative reverse transcription PCR

*C. thermocellum* WT and mutant strains were cultivated to mid-log phase at *t*_*max*_ for cellulose hydrolysis with Avicel as the carbon source (Table 1), and total RNAs were isolated by using an RNeasy Mini kit (Qiagen). Three independent replicates were performed for each strain to calculate the average values and standard deviations. Reverse transcription of the RNA was performed with SuperScript III First-Strand Synthesis Supermix (Invitrogen) and random hexamer primers. The resulting cDNA was used as a template for quantitative PCR with the primers listed in Additional file 2 using a Faststart Universal SYBR Green Master (Rox) kit (Roche). RNA samples were confirmed to be DNA-free by lack of PCR products when the reverse transcription step was omitted. Clo1313_2095 encoding a glyceraldehyde 3-phosphate dehydrogenase in *C. thermocellum* DSM1313 was used as a reference to calculate the relative gene expression levels [56].

### Preparation of cellulosomal proteins and extracellular proteins

Cellulosomes were prepared by a modified cellulose-affinity procedure [21]. WT and mutant strains were cultivated in GS-2 medium with 5 g/L Avicel as the sole carbon source for 3 to 13 days at 55°C until the late exponential phase, when cellulosomes were released into the medium. 200 mL of the culture supernatants were incubated at 4°C overnight with 20 mg phosphoric acid swollen amorphous cellulose and then centrifuged at 4,500 × g (Allegra X-22R, Beckman Coulter, Inc.) in a swinging-bucket rotor (SX4250) for 1 h at 4°C. The pellets were resuspended with 10 mL 50 mM Tris-HCl buffer (pH 7.0) containing 50 mM CaCl_2_ and 50 mM dithiothreitol, and then dialyzed against 1 liter sterile distilled water overnight. After dissolving the phosphoric acid swollen amorphous cellulose, the supernatants were concentrated to 0.5 to 1 mL by ultrafiltration (100 kD, Sartorius Stedim Biotech) and analyzed by SDS-PAGE.

To obtain extracellular proteins, 10 mL of the culture were centrifuged at 8,000 × g in a fixed angle rotor for 20 min to remove cells. The broth supernatant was condensed to 0.5 mL by ultrafiltration at 5,000 × g for 30 min, and the concentrated protein samples were used for SDS-PAGE analysis and protein quantification.

### Protein analysis methods

Sodium dodecyl sulfate-polyacrylamide gel electrophoresis (SDS-PAGE) was conducted with a 5% polyacrylamide stacking gel and 8% polyacrylamide resolving gel in a Mini-Protean II electrophoresis cell (Bio-Rad) [57]. Gels were either stained directly with Coomassie blue to visualize the proteins, or electroblotted for immunological detection [58]. The molecular weight of the protein was estimated based on the relative mobility of protein ladders (10 to 230 kDa, New England BioLabs).

For immunoblotting, the SDS-polyacrylamide gel was incubated in a standard transfer buffer (50 mM Tris-HCl, pH 8.3, 40 mM glycine, 0.1% SDS, and 20% methanol) for 30 min before wet blotting onto a presoaked polyvinylidene difluoride membrane at 200 mA for 2 h in an ice bath. The membrane was then blocked by incubating overnight in TBST buffer (20 mM Tris-HCl, 138 mM NaCl, 0.1% Tween 20, pH 7.6) containing 50 g/L skim milk and washed three times with TBST buffer. Afterwards, the membrane was incubated for 3 h with anti-XDocII peptide rabbit IgG (XDocII target peptide: HKHFGATSSDYDAQ) according to the manufacturer’s protocol (Sangon Biotech). Then, the membrane was washed three more times with TBST buffer, incubated for a further 3 h with a solution of anti-rabbit IgG(Fc) goat IgG conjugated with alkaline phosphatase (Promega), washed as before, and visualized with Western blue stabilized substrate (Promega) in accordance with manufacturers’ protocol.

For mass spectroscopy, individual protein bands were cut from an SDS-polyacrylamide gel stained with Coomassie blue, and the in-gel protein was washed, reduced, alkylated, and digested with trypsin, as described [27]. The resulting peptide mixture was extracted from the gel slice using 60% (vol/vol) acetonitrile in 0.1% (vol/vol) formic acid, dried by lyophilization, and dissolved in 0.1% (vol/vol) formic acid. The peptide samples were analyzed by an LTQ-ESI-MS/MS (Thermo Finnigan, San Jose, CA, USA), using a surveyor high performance liquid chromatography (HPLC) system equipped with a C18 RP column (0.18 × 100 mm, Thermo Electron Corporation) according to published protocol [59] with some modifications. In detail, the mobile phase A contained 0.1% (vol/vol) formic acid and 5% (vol/vol) acetonitrile in water, the mobile phase B contained 0.1% (vol/vol) formic acid in acetonitrile, and the peptide mixtures were eluted by using a gradient of 5 to 65% mobile phase B over 45 min at a flow rate of 500 nl/min. The temperature of the heated capillary was set at 200°C, the voltage applied to the ESI needle was 2.1 kV, and the normalized collision energy was 35. An initial full MS survey scan (about 10 ms) was performed for the *m*/*z* range 350 to 2,000, followed by several data-dependent scans, and the five most intense ions from the MS survey scan were subjected to five MS/MS scans. For protein identification, the MS spectra results were searched against the 2 August 2013 release of the *C. thermocellum* DSM1313 genome available at the NCBI website (NCBI reference sequence NC_017304.1) using the SEQUEST program from the Thermo Discoverer Proteome 1.0 database. The database was digested *in silico* with trypsin to include masses within the range of 350 to 3,500 Da, and a high-stringency filter was applied to the search results. Peptide ions with +1, +2, or +3 charges were accepted if they had a cross-correlation (Xcorr) score of at least 1.5, 2.5, and 3.5, respectively.

### Fermentation of *C. thermocellum* strains

Fermentation was performed in 200-mL glass bottles shaken at 170 rpm under anoxic conditions. *C. thermocellum* DSM1313 WT and mutant strains were grown in 100 mL GS-2 medium at 55°C with 5 or 10 g/L Avicel (Sigma PH101) as the sole carbon source, and three independent fermentations were done for each strain. Starting from the initial time point, 1.2-mL samples were taken every 8 to 24 h with a 2.5-mL syringe from each culture. 1 mL of each sample was transferred to another tube and centrifuged (8,000 × g, 10 min) at 4°C to separate the cells from the supernatant. The pellets were used to determine the amount of residual cellulose (see below), and the supernatants were used for analyses of fermentation products and proteins. The remaining parts of the samples (about 0.2 mL) from each of the three independent cultures of each strain were combined (0.6 mL), and 0.5 mL of this sample was transferred to another tube and centrifuged to pellet cells (8,000 × g, 10 min, 4°C). These combined pellets were used to determine the abundance of total cellular protein. For further analyses, the cell pellets were washed with sterile distilled water, and the pellets and supernatants were stored at -80°C.

The protein content of the supernatants was determined by the Bradford method [60] with a commercial protein quantification kit (BioMed Technology). Similarly, the total cell protein was determined by resuspending the pelleted cells in 0.5 mL 0.2 M NaOH and boiling for 10 min. 20 μl of the cleared supernatant obtained by centrifugation (8,000 × g, 10 min) was used directly for protein quantification.

### Determination of residual cellulose during fermentation

The residual cellulose in each sample was determined by a modified saccharification method [31]. Samples obtained by centrifugation and freeze drying overnight using an Alpha 1-2LP plus lyophilizer were incubated in 0.25 mL of 72% sulfuric acid for cellulose hydrolysis (30°C, 1 h). The hydrolysates were transferred to 20 mL Hungate tubes, and 4.75 mL sterile distilled water was added to reduce the acid concentration to 3.6%. The Hungate tubes were sealed with rubber stoppers to maintain pressure and autoclaved for 1 h (121°C) for dilute acid hydrolysis. Hydrolysate samples were further diluted and neutralized by adding 5 mL sterile distilled water and 0.42 g CaCO_3_ after the tubes cooled to room temperature. 2 mL of each sample were microfiltrated (polyethersulfone syringe filter with a pore size of 0.22 μm, ANPEL Scientific Instrument Co., Ltd.) and used for glucose quantification via HPLC (Aminex HPX-87H, 55°C, 0.5 mL/min) [61]. Glucose recovery standards were prepared and treated similarly to correct for sugar loss during dilute acid hydrolysis, as described [31]. The monomer mass of Avicel was assumed to be 162 g/mol with 5% moisture, based on which the residual Avicel was calculated and represented by glucose equivalents (mM).

### Calculation of cellulose hydrolysis rates

The cellulose consumption data were fitted to a sigmoidal curve based on a five-parameter Richards equation [32]:

$$y\left(t\right)=A_{0}+\frac{A_{t}‒A_{0}}{{\left(1+e^{\frac{t_{0}‒t}{\mathrm{sl}}}\right)}^{\mathrm{ap}}}$$

The parameters are defined as follows: *A*_*0*_ (lower horizontal value), *A*_*t*_ (higher horizontal value), *t*_*0*_ (inflection point), *sl* (slope at *t*_*0*_), and *ap* (asymmetry parameter), and the variable *t* is the cultivation time [17]. Curve fitting was performed with the Origin 8.5 software suite (OriginLab Corporation), and the adjusted R-square was calculated for curve evaluation (Additional file 10). The first derivative of the curve was defined to calculate the maximum hydrolysis rate (*V*_*max*_), while the second derivative of the curve was equal to 0 and used to calculate *t*_*max*_, that is, the cultivation time at *V*_*max*_[17].

### Scanning electron microscopy

Scanning electron microscopy was performed with *C. thermocellum* cells grown with cellobiose as the carbon source. 1 mL of broth (OD_600_ < 0.3) was collected by centrifugation (2,200 × g, 5 min), and the pelleted cells were resuspended in 2.5% glutaraldehyde in PBS buffer and incubated overnight at 4°C without shaking. Samples were washed with PBS buffer three times and incubated with osmium tetroxide (2%) for 1 h. The osmium-treated cells were washed with PBS buffer three times and incubated sequentially in PBS buffer with 30%, 50%, 70%, 90%, and 100% ethanol for 10 min each. Subsequently the cells were incubated in a 50:50 (v/v) solution of ethanol and tert-butyl alcohol for 10 min followed by incubation in 100% tert-butyl alcohol for 15 min. The samples were freeze dried until the tert-butyl alcohol was completely evaporated. The dried cells were mounted on a specimen stub using electrically conductive double-sided adhesive tape for gold alloy coating, and were observed using a field emission scanning electron microscope (S-4800, Hitachi).

### Cell-associated cellulose hydrolysis assay

The cell-associated cellulose hydrolysis activity of *C. themocellum* WT and mutant strains was determined under oxic conditions to exclude the influence of cell metabolism. All strains were cultivated with 5 g/L cellobiose as the carbon source until the early exponential phase (OD_600_ < 0.3). 40 mL of the culture was centrifuged at 4°C (2,200 × g, 10 min), and the pellets were washed once with 10 mL of reaction buffer (20 mM acetate, 10 mM CaCl_2_, 5 mM L-cysteine, and 2 mM EDTA, pH 5.0) [62]. Cells were collected by centrifugation (4°C, 2,200 × g, 10 min), resuspended in the reaction buffer, and normalized with respect to cell density. The reaction mixture contained 200 μl of cell suspension and 5 mg Avicel in 1 mL reaction buffer. The hydrolysis assay was performed by incubating the reactions at 55°C for 24 h on a rotary shaker at 170 rpm. Control reactions without Avicel or cell suspension were done in parallel. The reducing sugars produced in each sample were measured by the 3,5-dinitrosalicylic acid method [35].

### Cellulose-binding assay

The cellulose-binding assays were performed as described [63]. *C. thermocellum* strains were cultivated under anoxic conditions at 55°C with 5 g/L cellobiose as the carbon source until early exponential growth phase (OD_600_ < 0.3). For each sample, 3 mL of the culture was transferred to a 10-mL plastic tube together with a piece of filter paper (0.034 × 3 × 6.4 cm, Whatman). Tubes were shaken for 1 h at room temperature on a small platform shaker at 200 rpm, and the optical density at 600 nm from the supernatant (OD_*st*_) was measured by using a UV spectrophotometer. A sample without filter paper served as a control, and its optical density measurement (OD_*ct*_) was used to calculate the adhesion percentage following the formula (OD_*ct*_-OD_*st*_)/OD_*ct*_. To evaluate the potential of unspecific cell binding, cellulose adhesion was also measured with *E. coli* DH5 alpha cells.

## Abbreviations

CBM: carbohydrate-binding module; CipA: cellulosome-integrating protein A; CohI: type I cohesin; CohII: type II cohesin; DocI: type I dockerin; HPLC: high performance liquid chromatography; MS: mass spectroscopy; OD_600_: optical density at 600 nm; RT: reverse transcriptase; SDS-PAGE: sodium dodecyl sulfate-polyacrylamide gel electrophoresis; SLH: S-layer homology; TAIL-PCR: thermal asymmetric interlaced polymerase chain reaction; XDocII: X module linked with a type II dockerin.

## Competing interests

The authors declare that they have no competing interests.

## Authors’ contributions

WH, YF, GM, AML, YJL, and QC designed the research; WH, JZ, and GZC performed the experiments; WH, YF, GM, AML, YJL, and QC analyzed the data; and WH, YF, GM, AML, and YJL wrote the paper. All authors read and approved the final manuscript.

## Supplementary Material

Additional file 1 **Polymerase chain reaction analysis of the length of genes encoding CipA and OlpB in *C. thermocellum *** **strains DSM1313 and ATCC27405.** Click here for file

Additional file 2 Bacterial strains, plasmids, and oligonucleotides used in this study. Click here for file

Additional file 3 **Polymerase chain reaction and Southern hydridization analysis of the targetron insertions in the genes of *C. thermocellum *** **DSM1313.** Click here for file

Additional file 4 **Amino acid sequences of the CipA proteins of *C. thermocellum *** **strains.** Click here for file

Additional file 5 **Relative expression intensity of genes in the *cipA *** **operon assayed by qRT-PCR in CipA-truncated and secondary scaffoldin-disrupted mutants with Avicel as the carbon source.** Click here for file

Additional file 6 Mass spectroscopy analysis of CipA proteins in wild-type and CipA-ΔXDocII strains. Click here for file

Additional file 7 **CipA peptides in the wild-type and CipA-**Δ**XDocII strains identified by mass spectroscopy analysis.** Click here for file

Additional file 9 SDS-PAGE of supernatant proteins of wild-type and mutant strains. Click here for file

Additional file 8 Growth analysis of CipA-truncated and secondary scaffoldin-disrupted mutants with cellobiose as the carbon source. Click here for file

Additional file 11 **Quantification of polycellulosomal protuberances on the cell surfaces of wild-type *C. thermocellum *** **and secondary scaffoldin mutants.** Click here for file

Additional file 10 Curve-fitting parameters used to calculate the Avicel consumption data. Click here for file
