# Supplementary material for: The contribution of cellulosomal scaffoldins to cellulose hydrolysis by Clostridium thermocellum analyzed by using thermotargetrons
Source: Biotechnol Biofuels. 2014 May 29;7:80. doi: 10.1186/1754-6834-7-80 (PMC4045903; doi:10.1186/1754-6834-7-80)
Supplement: Additional file 1 — Polymerase chain reaction analysis of the length of genes encoding CipA and OlpB in C. thermocellum strains DSM1313 and ATCC27405. [file 1754-6834-7-80-S1.docx]

## Additional file 1. PCR analysis of the length of genes encoding CipA and OlpB in *C. thermocellum* strains DSM1313 and ATCC27405.

According to the genome annotation, CipA and OlpB of *C. thermocellum* DSM1313 contain 6 CohI and 4 CohII modules, respectively, whereas CipA and OlpB of the closely related strain *C. thermocellum* ATCC27405 are annotated as containing 9 CohI and 7 CohII modules, respectively [1]. PCRs with primer sets CipAup/CipAdown and OlpBup/OlpBdown flanking the relevant regions of *cipA* and *olpB* genes, respectively (Additional file 2, 3), were performed in parallel to investigate this difference. The PCR products of *olpB* of DSM1313 and ATCC27405 are the same size and thus likely to have similar numbers of CohII modules. The PCR products of CipA show a ladder of bands in both strains, which may be caused by the unspecific amplification due to repeated CohI sequences within CipA. The main band of DSM1313 is ~500 bp smaller than that of ATCC27405, and comigrates with the second band of the *cipA* ladder of ATCC27405. Thus, we infer that CipA of DSM1313 contains one less CohI module than that of ATCC27405. Taken together, these findings indicate that the functional domains of CipA and OlpB of DSM1313 were annotated incorrectly and actually contain 8 CohI modules and 7 CohII modules, respectively.

1. Feinberg L, Foden J, Barrett T, Davenport KW, Bruce D, Detter C, Tapia R, Han C, Lapidus A, Lucas S, et al: **Complete genome sequence of the cellulolytic thermophile *Clostridium thermocellum* DSM1313.** *J Bacteriol* 2011, **193:**2906-2907.
