# Supplementary material for: The contribution of cellulosomal scaffoldins to cellulose hydrolysis by Clostridium thermocellum analyzed by using thermotargetrons
Source: Biotechnol Biofuels. 2014 May 29;7:80. doi: 10.1186/1754-6834-7-80 (PMC4045903; doi:10.1186/1754-6834-7-80)
Supplement: Additional file 2 — Bacterial strains, plasmids, and oligonucleotides used in this study. [file 1754-6834-7-80-S2.docx]

Additional file 2

Bacterial strains and plasmids used in this study.

| Strains or Plasmids | Relevant characteristic | Reference or source | |
| --- | --- | --- | --- |
| **Strains** |  |  | |
| ***E. coli*** |  |  | |
| DH5α | F^−^*,* ϕ*80lacZΔM15, Δ(lacZYA-argF) U169, deoR, recA1, endA1, hsdR17(r_k_*^−^*, m_k_*^+^*), phoA, supE44,* λ^−^*, thi-1, gyrA96, relA1* | TransGene | |
| BL21(DE3) | F^−^*, ompT,* *gal,* *dcm,* *lon, hsdS_B_(r_B_*^−^ *m_B_^−^)* λ (DE3 [*lacI* lacUV5-T7 gene 1 *ind1* *sam7* *nin5*]) | TransGene | |
| ***C. thermocellum*** |  |  | |
| DSM1313 (WT)  ATCC27405 | Wild type stain  Wild type stain | DSMZ  ATCC | |
| CipA-ΔCBM-1  CipA-ΔCBM-2  CipA-Δ6CohI  CipA-ΔXDocII  ΔSdbA  ΔOrf2p  ΔOlpB  Δ7CohII  **Plasmids** | Derived from DSM1313, Clo1313_0627::CipA1158a  Derived from DSM1313, Clo1313_0627::CipA942a  Derived from DSM1313, Clo1313_0627::CipA1827s  Derived from DSM1313, Clo1313_0627::CipA3740s  Derived from DSM1313, Clo1313_0950::SdbA123s  Derived from DSM1313, Clo1313_0629::Orf2p_321s  Derived from DSM1313, Clo1313_0628::OlpB134s  Derived from DSM1313, Clo1313_1487::7CohII735a | This work  This work  [1]  This work  This work  This work  This work  This work | |
| pHK-TT1A  pHK-CipA942a  pHK-CipA1158a  pHK-CipA1827s  pHK-CipA3740s | *E. coli-C. thermocellum* shuttle vector, *groEl* promoter, Tel3c4c targetron cassette, Cm^R^/Tm^R^  Derived from pHK-TT1A, *E. coli-C. thermocellum* shuttle vector, *groEl* promoter, CipA942a intron, Cm^R^/Tm^R^  Derived from pHK-TT1A, *E. coli-C. thermocellum* shuttle vector, *groEl* promoter, CipA1158a intron, Cm^R^/Tm^R^  Derived from pHK-TT1A, *E. coli-C. thermocellum* shuttle vector, *groEl* promoter, CipA1827s intron, Cm^R^/Tm^R^  Derived from pHK-TT1A, *E. coli-C. thermocellum* shuttle vector, *groEl* promoter, CipA3740s intron, Cm^R^/Tm^R^ | [1]  This work  This work  [1]  This work |  |
| pHK-OlpB134s  pHK-Orf2p_321s  pHK-SdbA123s  pHK-7CohII735a | Derived from pHK-TT1A, *E. coli-C. thermocellum* shuttle vector, *groEl* promoter, OlpB134s intron, Cm^R^/Tm^R^  Derived from pHK-TT1A, *E. coli-C. thermocellum* shuttle vector, *groEl* promoter, Orf2p_321s intron, Cm^R^/Tm^R^  Derived from pHK-TT1A, *E. coli-C. thermocellum* shuttle vector, *groEl* promoter, SdbA123s intron, Cm^R^/Tm^R^  Derived from pHK-TT1A, *E. coli-C. thermocellum* shuttle vector, *groEl* promoter, 7CohII735a intron, Cm^R^/Tm^R^ | This work  This work  This work  This work |  |

Oligonucleotides used in this study.

| Primers and oligos | Sequences (5’-3’)^*^ | Notes |
| --- | --- | --- |
| TeI3cUNIV  CipA942a IBS12  CipA942a EBS2  CipA942a EBS1a  CipA1158a IBS12  CipA1158a EBS2  CipA1158a EBS1a  CipA1827s IBS12  CipA1827s EBS2  CipA1827s EBS1a  CipA3740s IBS12  CipA3740s EBS2  CipA3740s EBS1a  OlpB134s IBS12  OlpB134s EBS2  OlpB134s EBS1a  Orf2p_321s IBS12  Orf2p_321s EBS2  Orf2p_321s EBS1a  SdbA123s IBS12  SdbA123s EBS2  SdbA123s EBS1a  7CohII735a IBS12  7CohII735a EBS2  7CohII735a EBS1a | TAACGAGGCTTCTAGC  AAAACTAGTAACACCACCGTCTATGTGCGACGCGAAAGCTAG  CGCTAGAAGCCTCGTTATGGTGAGCAGGCCAAAGATGCTG  CCCCGTaCgCTGAGTCTATAGCAGcGtATCCAATCC  AAAACTAGTAACCTTGAACTGAGGGTGCGACGCGAAAGCTAG  CGCTAGAAGCCTCGTTACAAGGAGCAGGCCAAAGATGCTG  CCCCGTaCgCTGACTGAGGAGCAGCGTATCCAATCC  AAAACTAGTAAACCGGGAGAATTGGTGCGACGCGAAAGCTAG  CGCTAGAAGCCTCGTTACCGGTAGCAGGCCAAAGATGCTG  CCCCGTaCgCTGAGAATTGAGCAGcGtATCCAATCC  AAAACTAGTAACCGGAGTACCTGCGTGCGACGCGAAAGCTAG  CGCTAGAAGCCTCGTTATCCGGAGCAGGCCAAAGATGCTG  CCCCGTaCgCTGAACCTGCAGCAGcGtATCCAATCC  AAAACTAGTAACTGAAGTCCATGTGTGCGACGCGAAAGCTAG  CGCTAGAAGCCTCGTTATTCAGAGCAGGCCAAAGATGCTG  CCCCGTaCgCTGACCATGTAGCAGcGtATCCAATCC  AAAACTAGTAAGGGAGATCAGTACGTGCGACGCGAAAGCTAG  CGCTAGAAGCCTCGTTACTCCCAGCAGGCCAAAGATGCTG  CCCCGTaCgCTGACAGTACAGCAGcGtATCCAATCC  AAAACTAGTAAGTTTGACCGCAATGTGCGACGCGAAAGCTAG  CGCTAGAAGCCTCGTTACAAACAGCAGGCCAAAGATGCTG  CCCCGTaCgCTGACGCAATAGCAGcGtATCCAATCC  AAAACTAGTAAACTGGTATCCTGC GTGCGACGCGAAAGCTAG  CGCTAGAAGCCTCGTTACCAGTAGCAGGCCAAAGATGCTG  CCCCGTaCgCTGATCCTGCAGCAGcGtATCCAATCC | To construct  corresponding  targetrons [1] |
| Cipa942aF  Cipa942aR  CipA1158aF  CipA1158aR  CipA1827sF  CipA1827sR  CipA3740sF  CipA3740sR  OlpB134sF  OlpB134sR  Orf2p321sF  Orf2p321sR  SdbA123sF  SdbA123sR  7CohII735a-F  7CohII735a-R | GCAAAAATAAGAGCAACTG  TCATCTGTCGGTGTTGTTAC  GCAAAAATAAGAGCAACTG  TCATCTGTCGGTGTTGTTAC  GCAAAAATAAGAGCAACTG  TCATCTGTCGGTGTTGTTAC  GACCTTATCAATGGTGGAGTA  CGTCGTAATCACTTGATGTAG  GCTAATAATCTCTACCACATCC  ACCTTCGTCTTATCCAGTTC  CCACAGTCTATTCACTCCAA  GTCGCAACAATTACATCTCC  CAAGTTCTTCAACGCTCAAT  GCATTATCCAACACAGGTTC  TGGAACGGTGAGGTTATTAC  GTAGGCAGCAAGGTATGTAT | To confirm the insertion of corresponding targetrons at the desired sites |
| A-SP1  A-SP2  A-SP3  S-SP1  S-SP2  S-SP3 | AGGTTGGAGCCGAATCCGTGAGGA  ATGGTGAACGCAAGTGAAGTGACGCT  GATCTTGAGTCACTGGTGCGGTCAC  GTGACCGCACCAGTGACTCAAGATC  AGCGTCACTTCACTTGCGTTCACCAT  TCCTCACGGATTCGGCTCCAACCT | Specific primers for TAIL PCR |
| CipAup  CipAdown  OlpBup  OlpBdown | CAGCATACTTGAACGGTGTTCTTGT  CAACAGTAGCGTCGAAGGAGAAGT  TGTGCTATACATGAGCTGTTGAAGG  GCTGCTGCGTCAAAGAACTAAAC | To investigate CipA and OlpB genes in DSM1313 |
| Probe172-F  Probe172-R | GCAGGACATTCAACATCATC  GATACTCATCTACAGCCTCTC | To generate probe  for Southern blot  [1] |
| CipA-F  CipA-R  SdbA-F  SdbA-R  OlpB-F  OlpB-R  Orf2p-F  Orf2p-R  OlpA-F  OlpA-R  gapDH-F  gapDH-R | CAGCATACTTGAACGGTGTTCTTGT  TCATCTGACGGCGGTATTGTTGT  CCACGCCGTCAGTTACTCCTTC  CATCTATTGCCGCCTTTGCTTCTC  GACGACTGCGGTAGCGAATGAT  CTCCTCTGCCACACCTGTCTCT  AACAACAACAGCACCATCACAGAC  GGTGTCGGTGAAGCTGAAGGATTA  AGGTGGCGGTACGGGTTCTT  ATGCGGCTGCTCAGTTGTCTT  AGCAGCAGGCAACATCATTCC  ACGGCAAGTCAAGTCAACAACA | For qRT-PCR |

* Mutations are indicated by lower case letters. Restriction sites introduced by the oligonucleotides are underlined.

1. Mohr G, Hong W, Zhang J, Cui GZ, Yang Y, Cui Q, Liu YJ, Lambowitz AM: **A targetron system for gene targeting in thermophiles and its application in *Clostridium thermocellum*.** *PLoS One* 2013, **8:**e69032.
