# Supplementary material for: The contribution of cellulosomal scaffoldins to cellulose hydrolysis by Clostridium thermocellum analyzed by using thermotargetrons
Source: Biotechnol Biofuels. 2014 May 29;7:80. doi: 10.1186/1754-6834-7-80 (PMC4045903; doi:10.1186/1754-6834-7-80)
Supplement: Additional file 3 — Polymerase chain reaction and Southern hydridization analysis of the targetron insertions in the genes of C. thermocellum DSM1313. [file 1754-6834-7-80-S3.docx]

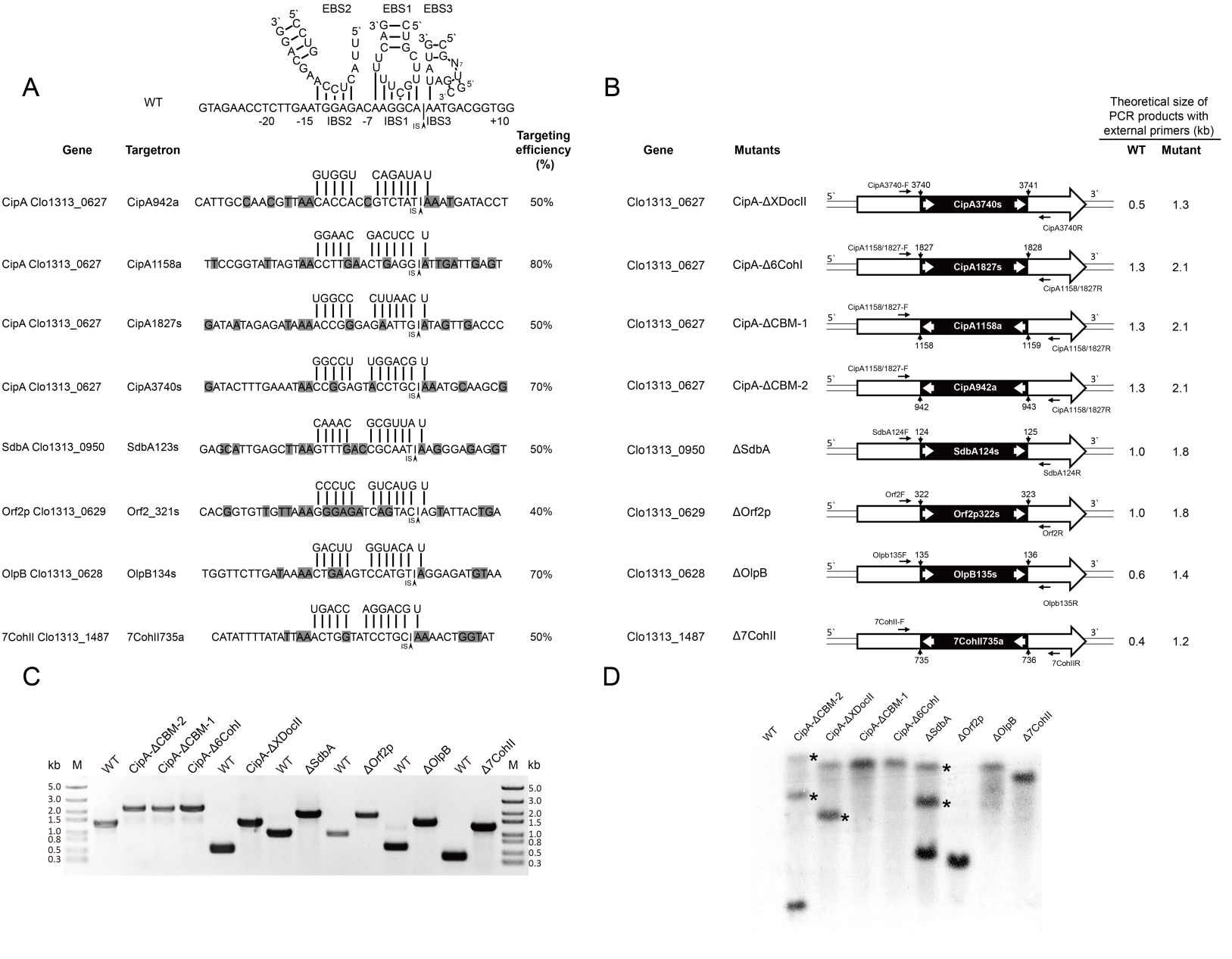


Additional file 3. Construction of thermotargetrons for *C. thermocellum* DSM1313 and confirmation of targetron insertion by PCR and Southern hybridization analysis.

(A) Schematic representation of the construction of thermotargetrons for *C. thermocellum* DSM1313. Target sites, EBS/IBS base-pairing interactions, and targeting efficiencies for eight constructed targetrons are shown. Gray shading highlights nucleotide residues in the *C. thermocellum* target sites that match those in the wild-type target site, which is shown above for comparison. The arrowhead indicates the intron-insertion site (IS). The targeting efficiency was calculated as the percentage of thiamphenicol-resistant transformants in which the disruption of the target gene was confirmed by colony PCR and sequencing [1]. (B) Schematic representation of the targetron insertions. Open arrows show the orientation of the target gene with the direction of the arrow indicating 5’ to 3’. Black boxes represent the inserted targetrons, whose insertion sites are indicated by arrowheads with nucleotide position numbers. The direction of the targetron insertion is represented by the white arrowheads within the targetron boxes. Primers used to analyze the targetron insertion are indicated by horizontal arrows. The expected sizes of the PCR products of wild type (WT) and disrupted genes are indicated to the right. (C) Colony PCR analysis of the targetron insertions in chromosomal DNA. WT, *C. thermocellum* DSM1313, M, DNA marker. The sequences of the primers used for colony PCR are listed in Additional file 2. (D) Southern hydridization analysis of targetron insertions in *C. thermocellum* mutant strains. Genomic DNAs isolated from wild-type and mutant cells were digested with BamHI and EcoRI at 37°C overnight (both enzymes do not cleave within the targetron). The digests were run in a 0.8% agarose gel, and blotted to a Nylon membrane (Hybond-NX, GE Healthcare), which was hybridized with intron probes generated with primers Probe172-F and Probe172-R (Additional file 2). A single band hybridizing to the intron probe indicates that the targtron integrated at a single position in the genome, whereas multiple bands indicate that the targetron integrated at more than one site [1]. Single bands of the size expected for integration at the desired target site were detected in CipA-ΔCBM-1, CipA-Δ6CohI, ΔOrf2p, ΔOlpB, and Δ7CohII shown here and previously in CipA-Δ6CohI [1]. Additional off-target insertions, indicated by asterisks, are detected in CipA-ΔCBM-2, CipA-ΔXDocII, and ΔSdbA. TAIL PCR analysis indicated that the off-target insertion site in CipA-ΔXDocII is in Clo1313_1971, encoding a dockerin containing protein, whose expression level is low and unchanged during growth with cellulose as carbon source [2]. This mutant has near wild-type rates of cellulose hydrolysis, suggesting little if any effect of the off-target integration. The two off-target integrations in CipA-ΔCBM-2 are in Clo1313_2646 (hypothetical protein) and Clo1313_1129 ([spore coat protein](http://www.ncbi.nlm.nih.gov/protein/ADU74193.1)), while those in ΔSdbA are in Clo1313_1277 (hypothetical protein) and Clo1313_1305 (GH10 xylanase). No expression of Clo1313_1305 (GH10 xylanase) is detected in Avicel-grown cells [3]. Thus, these genes are not expected to strongly affect cellulose hydrolysis. M, DNA marker.

1. Mohr G, Hong W, Zhang J, Cui GZ, Yang Y, Cui Q, Liu YJ, Lambowitz AM: **A targetron system for gene targeting in thermophiles and its application in *Clostridium thermocellum*.** *PLoS One* 2013, **8:**e69032.

2. Raman B, McKeown CK, Rodriguez M, Jr., Brown SD, Mielenz JR: **Transcriptomic analysis of *Clostridium thermocellum* ATCC 27405 cellulose fermentation.** *BMC Microbiol* 2011, **11:**134.

3. Gold ND, Martin VJ: **Global view of the *Clostridium thermocellum* cellulosome revealed by quantitative proteomic analysis.** *J Bacteriol* 2007, **189:**6787-6795.
