# Supplementary material for: The contribution of cellulosomal scaffoldins to cellulose hydrolysis by Clostridium thermocellum analyzed by using thermotargetrons
Source: Biotechnol Biofuels. 2014 May 29;7:80. doi: 10.1186/1754-6834-7-80 (PMC4045903; doi:10.1186/1754-6834-7-80)
Supplement: Additional file 4 — Amino acid sequences of the CipA proteins of C. thermocellum strains. [file 1754-6834-7-80-S4.pdf]

```
ATCC27405_CipA              MRKVISMLLVVAMLTTIFAAMIPQTVSAATMTVEIGKVTAAVGSKVEIPITLKGVPSKGMANCDFVLGYDPNVLEVTEVKPGSIIKDPDPSKSFDSAIYP   100
DSM1313(WT)_CipA            MRKVISMLLVVAMLTTIFAAMIPQTVSAATMTVEIGKVTAAVGSKVEIPITLKGVPSKGMANCDFVLGYDPNVLEVTEVKPGSIIKDPDPSKSFDSAIYP   100
CipA-ΔXDocII(CipA3740s)     MRKVISMLLVVAMLTTIFAAMIPQTVSAATMTVEIGKVTAAVGSKVEIPITLKGVPSKGMANCDFVLGYDPNVLEVTEVKPGSIIKDPDPSKSFDSAIYP   100
CipA-Δ6CohI(CipA1827s)      MRKVISMLLVVAMLTTIFAAMIPQTVSAATMTVEIGKVTAAVGSKVEIPITLKGVPSKGMANCDFVLGYDPNVLEVTEVKPGSIIKDPDPSKSFDSAIYP   100
CipA-ΔCBM-1(CipA1158a)      MRKVISMLLVVAMLTTIFAAMIPQTVSAATMTVEIGKVTAAVGSKVEIPITLKGVPSKGMANCDFVLGYDPNVLEVTEVKPGSIIKDPDPSKSFDSAIYP   100
CipA-ΔCBM-2(CipA942a)       MRKVISMLLVVAMLTTIFAAMIPQTVSAATMTVEIGKVTAAVGSKVEIPITLKGVPSKGMANCDFVLGYDPNVLEVTEVKPGSIIKDPDPSKSFDSAIYP   100

ATCC27405_CipA              DRKMIVFLFAEDSGRGTYAITQDGVFATIVATVKSAAAAPITLLEVGAFADNDLVEISTTFVAGGVNLGSSVPTTQPNVPSDGVVVEIGKVTGSVGTTVE   200
DSM1313(WT)_CipA            DRKMIVFLFAEDSGRGTYAITQDGVFATIVATVKSAAAAPITLLEVGAFADNDLVEISTTFVAGGVNLGSSVPTTQPNVPSDGVVVEIGKVTGSVGTTVE   200
CipA-ΔXDocII(CipA3740s)     DRKMIVFLFAEDSGRGTYAITQDGVFATIVATVKSAAAAPITLLEVGAFADNDLVEISTTFVAGGVNLGSSVPTTQPNVPSDGVVVEIGKVTGSVGTTVE   200
CipA-Δ6CohI(CipA1827s)      DRKMIVFLFAEDSGRGTYAITQDGVFATIVATVKSAAAAPITLLEVGAFADNDLVEISTTFVAGGVNLGSSVPTTQPNVPSDGVVVEIGKVTGSVGTTVE   200
CipA-ΔCBM-1(CipA1158a)      DRKMIVFLFAEDSGRGTYAITQDGVFATIVATVKSAAAAPITLLEVGAFADNDLVEISTTFVAGGVNLGSSVPTTQPNVPSDGVVVEIGKVTGSVGTTVE   200
CipA-ΔCBM-2(CipA942a)       DRKMIVFLFAEDSGRGTYAITQDGVFATIVATVKSAAAAPITLLEVGAFADNDLVEISTTFVAGGVNLGSSVPTTQPNVPSDGVVVEIGKVTGSVGTTVE   200

ATCC27405_CipA              IPVYFRGVPSKGIANCDFVFRYDPNVLEIIGIDPGDIIVDPNPTKSFDTAIYPDRKIIVFLFAEDSGTGAYAITKDGVFAKIRATVKSSAPGYITFDEVG   300
DSM1313(WT)_CipA            IPVYFRGVPSKGIANCDFVFRYDPNVLEIIGIDPGDIIVDPNPTKSFDTAIYPDRKIIVFLFAEDSGTGAYAITKDGVFAKIRATVKSSAPGYITFDEVG   300
CipA-ΔXDocII(CipA3740s)     IPVYFRGVPSKGIANCDFVFRYDPNVLEIIGIDPGDIIVDPNPTKSFDTAIYPDRKIIVFLFAEDSGTGAYAITKDGVFAKIRATVKSSAPGYITFDEVG   300
CipA-Δ6CohI(CipA1827s)      IPVYFRGVPSKGIANCDFVFRYDPNVLEIIGIDPGDIIVDPNPTKSFDTAIYPDRKIIVFLFAEDSGTGAYAITKDGVFAKIRATVKSSAPGYITFDEVG   300
CipA-ΔCBM-1(CipA1158a)      IPVYFRGVPSKGIANCDFVFRYDPNVLEIIGIDPGDIIVDPNPTKSFDTAIYPDRKIIVFLFAEDSGTGAYAITKDGVFAKIRATVKSSAPGYITFDEVG   300
CipA-ΔCBM-2(CipA942a)       IPVYFRGVPSKGIANCDFVFRYDPNVLEIIGIDPGDIIVDPNPTKSFDTAIYPDRKIIVFLFAEDSGTGAYAITKDGVFAKIRATVKSSAPGYITFDEVG   300

ATCC27405_CipA              GFADNDLVEQKVSFIDGGVNVGNATPTKGATPTNTATPTKSATATPTRPSVPTNTPTNTPANTPVSGNLKVEFYNSNPSDTTNSINPQFKVTNTGSSAID   400
DSM1313(WT)_CipA            GFADNDLVEQKVSFIDGGVNVGNATPTKGATPTNTATPTKSATATPTRPSVPTNTPTNTPANTPVSGNLKVEFYNSNPSDTTNSINPQFKVTNTGSSAID   400
CipA-ΔXDocII(CipA3740s)     GFADNDLVEQKVSFIDGGVNVGNATPTKGATPTNTATPTKSATATPTRPSVPTNTPTNTPANTPVSGNLKVEFYNSNPSDTTNSINPQFKVTNTGSSAID   400
CipA-Δ6CohI(CipA1827s)      GFADNDLVEQKVSFIDGGVNVGNATPTKGATPTNTATPTKSATATPTRPSVPTNTPTNTPANTPVSGNLKVEFYNSNPSDTTNSINPQFKVTNTGSSAID   400
CipA-ΔCBM-1(CipA1158a)      GFADNDLVEQKVSFIDGGVNVGNATPTKGATPTNTATPTKSATATPTRPSVPTNTPTNTPANTPVSGNLKVEFYNSNPSDTTNSINvg*..........   388
CipA-ΔCBM-2(CipA942a)       GFADNDLVEQKVSFvg*.....................................................................................   316
                                          ▲                                                                        ▲

ATCC27405_CipA              LSKLTLRYYYTVDGQKDQTFWCDHAAIIGSNGSYNGITSNVKGTFVKMSSSTNNADTYLEISFTGGTLEPGAHVQIQGRFAKNDWSNYTQSNDYSFKSAS   500
DSM1313(WT)_CipA            LSKLTLRYYYTVDGQKDQTFWCDHAAIIGSNGSYNGITSNVKGTFVKMSSSTNNADTYLEISFTGGTLEPGAHVQIQGRFAKNDWSNYTQSNDYSFKSAS   500
CipA-ΔXDocII(CipA3740s)     LSKLTLRYYYTVDGQKDQTFWCDHAAIIGSNGSYNGITSNVKGTFVKMSSSTNNADTYLEISFTGGTLEPGAHVQIQGRFAKNDWSNYTQSNDYSFKSAS   500
CipA-Δ6CohI(CipA1827s)      LSKLTLRYYYTVDGQKDQTFWCDHAAIIGSNGSYNGITSNVKGTFVKMSSSTNNADTYLEISFTGGTLEPGAHVQIQGRFAKNDWSNYTQSNDYSFKSAS   500
CipA-ΔCBM-1(CipA1158a)      ....................................................................................................   388
CipA-ΔCBM-2(CipA942a)       ....................................................................................................   316

                                    CipAup
                                    ──►
ATCC27405_CipA              QFVEWDQVTAYLNGVLVWGKEPGGSVVPSTQPVTTPPATTKPPATTKPPATTIPPSDDPNAIKIKVDTVNAKPGDTVNIPVRFSGIPSKGIANCDFVYSY   600
DSM1313(WT)_CipA            QFVEWDQVTAYLNGVLVWGKEPGGSVVPSTQPVTTPPATTKP......PATTIPPSDDPNAIKIKVDTVNAKPGDTVNIPVRFSGIPSKGIANCDFVYSY   594
CipA-ΔXDocII(CipA3740s)     QFVEWDQVTAYLNGVLVWGKEPGGSVVPSTQPVTTPPATTKP......PATTIPPSDDPNAIKIKVDTVNAKPGDTVNIPVRFSGIPSKGIANCDFVYSY   594
CipA-Δ6CohI(CipA1827s)      QFVEWDQVTAYLNGVLVWGKEPGGSVVPSTQPVTTPPATTKP......PATTIPPSDDPNAIKIKVDTVNAKPGDTVNIPVRFSGIPSKGIANCDFVYSY   594
CipA-ΔCBM-1(CipA1158a)      ....................................................................................................   388
CipA-ΔCBM-2(CipA942a)       ....................................................................................................   316

ATCC27405_CipA              DPNVLEIIEIKPGELIVDPNPDKSFDTAVYPDRKIIVFLFAEDSGTGAYAITKDGVFATIVAKVKSGAPNGLSVIKFVEVGGFANNDLVEQRTQFFDGGV   700
DSM1313(WT)_CipA            DPNVLEIIEIKPGELIVDPNPDKSFDTAVYPDRKIIVFLFAEDSGTGAYAITKDGVFATIVAKVKSGAPNGLSVIKFVEVGGFANNDLVEQRTQFFDGGV   694
CipA-ΔXDocII(CipA3740s)     DPNVLEIIEIKPGELIVDPNPDKSFDTAVYPDRKIIVFLFAEDSGTGAYAITKDGVFATIVAKVKSGAPNGLSVIKFVEVGGFANNDLVEQRTQFFDGGV   694
CipA-Δ6CohI(CipA1827s)      DPNVLEIIEIKPGELyrres*...............................................................................   614
CipA-ΔCBM-1(CipA1158a)      ....................................................................................................   388
CipA-ΔCBM-2(CipA942a)       ....................................................................................................   316
                                           ▲

ATCC27405_CipA              NVGDTTVPTTPTTPVTTPTDDSNAVRIKVDTVNAKPGDTVRIPVRFSGIPSKGIANCDFVYSYDPNVLEIIEIEPGDIIVDPNPDKSFDTAVYPDRKIIV   800
DSM1313(WT)_CipA            NVGDTTVPTTPTTPVTTPTDDSNAVRIKVDTVNAKPGDTVRIPVRFSGIPSKGIANCDFVYSYDPNVLEIIEIEPGDIIVDPNPDKSFDTAVYPDRKIIV   794
CipA-ΔXDocII(CipA3740s)     NVGDTTVPTTPTTPVTTPTDDSNAVRIKVDTVNAKPGDTVRIPVRFSGIPSKGIANCDFVYSYDPNVLEIIEIEPGDIIVDPNPDKSFDTAVYPDRKIIV   794
CipA-Δ6CohI(CipA1827s)      ....................................................................................................   614
CipA-ΔCBM-1(CipA1158a)      ....................................................................................................   388
CipA-ΔCBM-2(CipA942a)       ....................................................................................................   316

ATCC27405_CipA              FLFAEDSGTGAYAITKDGVFATIVAKVKSGAPNGLSVIKFVEVGGFANNDLVEQKTQFFDGGVNVGDTTEPATPTTPVTTPTTTDDLDAVRIKVDTVNAK   900
DSM1313(WT)_CipA            FLFAEDSGTGAYAITKDGVFATIVAKVKSGAPNGLSVIKFVEVGGFANNDLVEQKTQFFDG.......................................   855
CipA-ΔXDocII(CipA3740s)     FLFAEDSGTGAYAITKDGVFATIVAKVKSGAPNGLSVIKFVEVGGFANNDLVEQKTQFFDG.......................................   855
CipA-Δ6CohI(CipA1827s)      ....................................................................................................   614
CipA-ΔCBM-1(CipA1158a)      ....................................................................................................   388
CipA-ΔCBM-2(CipA942a)       ....................................................................................................   316

ATCC27405_CipA              PGDTVRIPVRFSGIPSKGIANCDFVYSYDPNVLEIIEIEPGDIIVDPNPDKSFDTAVYPDRKIIVFLFAEDSGTGAYAITKDGVFATIVAKVKSGAPNGL  1000
DSM1313(WT)_CipA            ....................................................................................................   855
CipA-ΔXDocII(CipA3740s)     ....................................................................................................   855
CipA-Δ6CohI(CipA1827s)      ....................................................................................................   614
CipA-ΔCBM-1(CipA1158a)      ....................................................................................................   388
CipA-ΔCBM-2(CipA942a)       ....................................................................................................   316

ATCC27405_CipA              SVIKFVEVGGFANNDLVEQKTQFFDGGVNVGDTTEPATPTTPVTTPTTTDDLDAVRIKVDTVNAKPGDTVRIPVRFSGIPSKGIANCDFVYSYDPNVLEI  1100
DSM1313(WT)_CipA            ....................................................................................................   855
CipA-ΔXDocII(CipA3740s)     ....................................................................................................   855
CipA-Δ6CohI(CipA1827s)      ....................................................................................................   614
CipA-ΔCBM-1(CipA1158a)      ....................................................................................................   388
CipA-ΔCBM-2(CipA942a)       ....................................................................................................   316

ATCC27405_CipA              IEIEPGDIIVDPNPDKSFDTAVYPDRKIIVFLFAEDSGTGAYAITKDGVFATIVAKVKEGAPNGLSVIKFVEVGGFANNDLVEQKTQFFDGGVNVGDTTE  1200
DSM1313(WT)_CipA            ....................................................................................................   855
CipA-ΔXDocII(CipA3740s)     ....................................................................................................   855
CipA-Δ6CohI(CipA1827s)      ....................................................................................................   614
CipA-ΔCBM-1(CipA1158a)      ....................................................................................................   388
CipA-ΔCBM-2(CipA942a)       ....................................................................................................   316

ATCC27405_CipA              PATPTTPVTTPTTTDDLDAVRIKVDTVNAKPGDTVRIPVRFSGIPSKGIANCDFVYSYDPNVLEIIEIEPGELIVDPNPTKSFDTAVYPDRKMIVFLFAE  1300
DSM1313(WT)_CipA            ....................................................................................................   855
CipA-ΔXDocII(CipA3740s)     ....................................................................................................   855
CipA-Δ6CohI(CipA1827s)      ....................................................................................................   614
CipA-ΔCBM-1(CipA1158a)      ....................................................................................................   388
CipA-ΔCBM-2(CipA942a)       ....................................................................................................   316

ATCC27405_CipA              DSGTGAYAITEDGVFATIVAKVKSGAPNGLSVIKFVEVGGFANNDLVEQKTQFFDGGVNVGDTTEPATPTTPVTTPTTTDDLDAVRIKVDTVNAKPGDTV  1400
DSM1313(WT)_CipA            ..............................................................GVNVGDTTEPATPTTPVTTPTTTDDLDAVRIKVDTVNAKPGDTV   899
CipA-ΔXDocII(CipA3740s)     ..............................................................GVNVGDTTEPATPTTPVTTPTTTDDLDAVRIKVDTVNAKPGDTV   899
CipA-Δ6CohI(CipA1827s)      ....................................................................................................   614
CipA-ΔCBM-1(CipA1158a)      ....................................................................................................   388
CipA-ΔCBM-2(CipA942a)       ....................................................................................................   316

ATCC27405_CipA              RIPVRFSGIPSKGIANCDFVYSYDPNVLEIIEIEPGDIIVDPNPDKSFDTAVYPDRKIIVFLFAEDSGTGAYAITKDGVFATIVAKVKEGAPNGLSVIKF  1500
DSM1313(WT)_CipA            RIPVRFSGIPSKGIANCDFVYSYDPNVLEIIEIEPGDIIVDPNPTKSFDTAVYPDRKIIVFLFAEDSGTGAYAITKDGVFATIVAKVKEGAPNGLSVIKF   999
CipA-ΔXDocII(CipA3740s)     RIPVRFSGIPSKGIANCDFVYSYDPNVLEIIEIEPGDIIVDPNPTKSFDTAVYPDRKIIVFLFAEDSGTGAYAITKDGVFATIVAKVKEGAPNGLSVIKF   999
CipA-Δ6CohI(CipA1827s)      ....................................................................................................   614
CipA-ΔCBM-1(CipA1158a)      ....................................................................................................   388
CipA-ΔCBM-2(CipA942a)       ....................................................................................................   316

ATCC27405_CipA              VEVGGFANNDLVEQKTQFFDGGVNVGDTTVPTTSPTTTPPEPTITPNKLTLKIGRAEGRPGDTVEIPVNLYGVPQKGIASGDFVVSYDPNVLEIIEIEPG  1600
DSM1313(WT)_CipA            VEVGGFANNDLVEQKTQFFDGGVNVGDTTVPTTSPTTTPPEPTITPNKLTLKIGRAEGRPGDTVEIPVNLYGVPQKGIASGDFVVSYDPNVLEIIEIEPG  1099
CipA-ΔXDocII(CipA3740s)     VEVGGFANNDLVEQKTQFFDGGVNVGDTTVPTTSPTTTPPEPTITPNKLTLKIGRAEGRPGDTVEIPVNLYGVPQKGIASGDFVVSYDPNVLEIIEIEPG  1099
CipA-Δ6CohI(CipA1827s)      ....................................................................................................   614
CipA-ΔCBM-1(CipA1158a)      ....................................................................................................   388
CipA-ΔCBM-2(CipA942a)       ....................................................................................................   316

ATCC27405_CipA              ELIVDPNPTKSFDTAVYPDRKMIVFLFAEDSGTGAYAITEDGVFATIVAKVKEGAPEGFSAIEISEFGAFADNDLVEVETDLINGGVLVTNKPVIEGYKV  1700
DSM1313(WT)_CipA            ELIVDPNPTKSFDTAVYPDRKMIVFLFAEDSGTGAYAITEDGVFATIVAKVKEGAPEGFSAIEISEFGAFADNDLVEVETDLINGGVLVTNKPVIEGYKV  1199
CipA-ΔXDocII(CipA3740s)     ELIVDPNPTKSFDTAVYPDRKMIVFLFAEDSGTGAYAITEDGVFATIVAKVKEGAPEGFSAIEISEFGAFADNDLVEVETDLINGGVLVTNKPVIEGYKV  1199
CipA-Δ6CohI(CipA1827s)      ....................................................................................................   614
CipA-ΔCBM-1(CipA1158a)      ....................................................................................................   388
CipA-ΔCBM-2(CipA942a)       ....................................................................................................   316

                                         CipAdown
                                         ◄──
ATCC27405_CipA              SGYILPDFSFDATVAPLVKAGFKVEIVGTELYAVTDANGYFEITGVPANASGYTLKISRATYLDRVIANVVVTGDTSVSTSQAPIMMWVGDIVKDNSINL  1800
DSM1313(WT)_CipA            SGYILPDFSFDATVAPLVKAGFKVEIVGTELYAVTDANGYFEITGVPANASGYTLKISRATYLDRVIANVVVTGDTSVSTSQAPIMMWVGDIVKDNSINL  1299
CipA-ΔXDocII(CipA3740s)     SGYILPDFSFDATVAPLVKAGFKVEIVGTELYAVTDANGYFEITGVPAcdakasqmivplaqqartgpvvppiva*........................  1274
CipA-Δ6CohI(CipA1827s)      ....................................................................................................   614
CipA-ΔCBM-1(CipA1158a)      ....................................................................................................   388
CipA-ΔCBM-2(CipA942a)       ....................................................................................................   316
                                                                                ▲

ATCC27405_CipA              LDVAEVIRCFNATKGSANYVEELDINRNGAINMQDIMIVHKHFGATSSDYDA*                                                1852
DSM1313(WT)_CipA            LDVAEVIRCFNATKGSANYVEELDINRNGAINMQDIMIV[HKHFGATSSDYDA]*                                              1351
CipA-ΔXDocII(CipA3740s)     .....................................................                                                1274
CipA-Δ6CohI(CipA1827s)      .....................................................                                                 614
CipA-ΔCBM-1(CipA1158a)      .....................................................                                                 388
CipA-ΔCBM-2(CipA942a)       .....................................................                                                 316
```

Additional file 4. Amino acid sequences of the CipA proteins of *C. thermocellum* strains.

The wild-type CipA sequences are from the genome sequence of *C. thermocellum* ATCC27405 (accession number YP_001039466) and *C. thermocellum* DSM 1313 (accession number ADU73707), which may contain two more CohI modules according to our PCR analysis (Additional file 1). The horizontal arrows ahead of amino acids 509-517 and amino acids 1715-1723 indicate the binding sites of the PCR primers used to investigate the difference between *cipA* genes of ATCC27405 and DSM1313 (Additional file 1 and 2). Black triangles indicate the insertion sites of the targetrons, and the amino acids derived from intron sequences in frame with *cipA* are in lower case. The XDocII target peptide sequence at the C-terminus of CipA of WT is framed in a black box.
