# Supplementary material for: The contribution of cellulosomal scaffoldins to cellulose hydrolysis by Clostridium thermocellum analyzed by using thermotargetrons
Source: Biotechnol Biofuels. 2014 May 29;7:80. doi: 10.1186/1754-6834-7-80 (PMC4045903; doi:10.1186/1754-6834-7-80)
Supplement: Additional file 5 — Relative expression intensity of genes in the cipA operon assayed by qRT-PCR in CipA-truncated and secondary scaffoldin-disrupted mutants with Avicel as the carbon source. [file 1754-6834-7-80-S5.docx]

## Additional file 5. Relative expression intensity of genes in the *cipA* operon assayed by qRT-PCR in CipA-truncated and secondary scaffoldin-disrupted mutants with Avicel as carbon source.

Three independent replicates were performed for each strain. The bar graphs show the average value, with the standard deviation indicated by the error bars. All strains were cultivated to mid-log phase for total RNA isolation. Clo1313_2095 encoding a glyceraldehyde 3-phosphate dehydrogenase in *C. thermocellum* DSM1313 was used as a reference to calculate the relative expression levels of genes in the *cipA* operon (*cipA*, *olpB*, *orf2p* and *olpA*) and *sdbA*. The effect of gene disruption by targetron insertion was determined by dividing the relative amount of transcript of downstream genes by that of *cipA* in the same strain. In comparison with the wild-type strain (WT), the targetron insertion in CipA-ΔXDocII did not decrease but increased the relative level of OlpB, Orf2p and OlpA transcripts.
