# Supplementary material for: The contribution of cellulosomal scaffoldins to cellulose hydrolysis by Clostridium thermocellum analyzed by using thermotargetrons
Source: Biotechnol Biofuels. 2014 May 29;7:80. doi: 10.1186/1754-6834-7-80 (PMC4045903; doi:10.1186/1754-6834-7-80)
Supplement: Additional file 6 — Mass spectroscopy analysis of CipA proteins in wild-type and CipA-ΔXDocII strains. [file 1754-6834-7-80-S6.docx]

Additional file 6: Mass spectroscopy analysis of CipA proteins in wild-type and CipA-ΔXDocII strains

| Strains | Protein | Accession No. | Coverage (%)^1^ | No. of peptide ions | No. of amino acids | Mol weight (kDa) | Xcorr Score^2^ | Reference |
| --- | --- | --- | --- | --- | --- | --- | --- | --- |
| WT | CipA | gi125975556 | 61.54 | 474 | 1841 | 196 | 1881 | [1] |
|  | CipA (Partial) | gi489618342 | 30.2 | 60 | 841 | 91 | 252 | [2] |
| CipA-ΔXDocII | CipA | gi125975556 | 50.57 | 221 | 1841 | 196 | 892 | [1] |

^1^ Percentage of amino acid coverage to the matched protein, and covered amino acids are highlighted by green shading in Additional file 7.

^2^ Only the proteins with a cross correlation (Xcorr) score of >100 are listed.

1. Wu JD, Orme-Johnson WH, Demain AL: **Two components of an extracellular protein aggregate of *Clostridium thermocellum* together degrade crystalline cellulose.** *Biochemistry* 1988, **27:**1703-1709.

2. Currie DH, Herring CD, Guss AM, Olson DG, Hogsett DA, Lynd LR: **Functional heterologous expression of an engineered full length CipA from *Clostridium thermocellum* in *Thermoanaerobacterium saccharolyticum*.** *Biotechnology for biofuels* 2013, **6:**32.
