# Supplementary material for: The contribution of cellulosomal scaffoldins to cellulose hydrolysis by Clostridium thermocellum analyzed by using thermotargetrons
Source: Biotechnol Biofuels. 2014 May 29;7:80. doi: 10.1186/1754-6834-7-80 (PMC4045903; doi:10.1186/1754-6834-7-80)
Supplement: Additional file 7 — CipA peptides in the wild-type and CipA-ΔXDocII strains identified by mass spectroscopy analysis. [file 1754-6834-7-80-S7.docx]

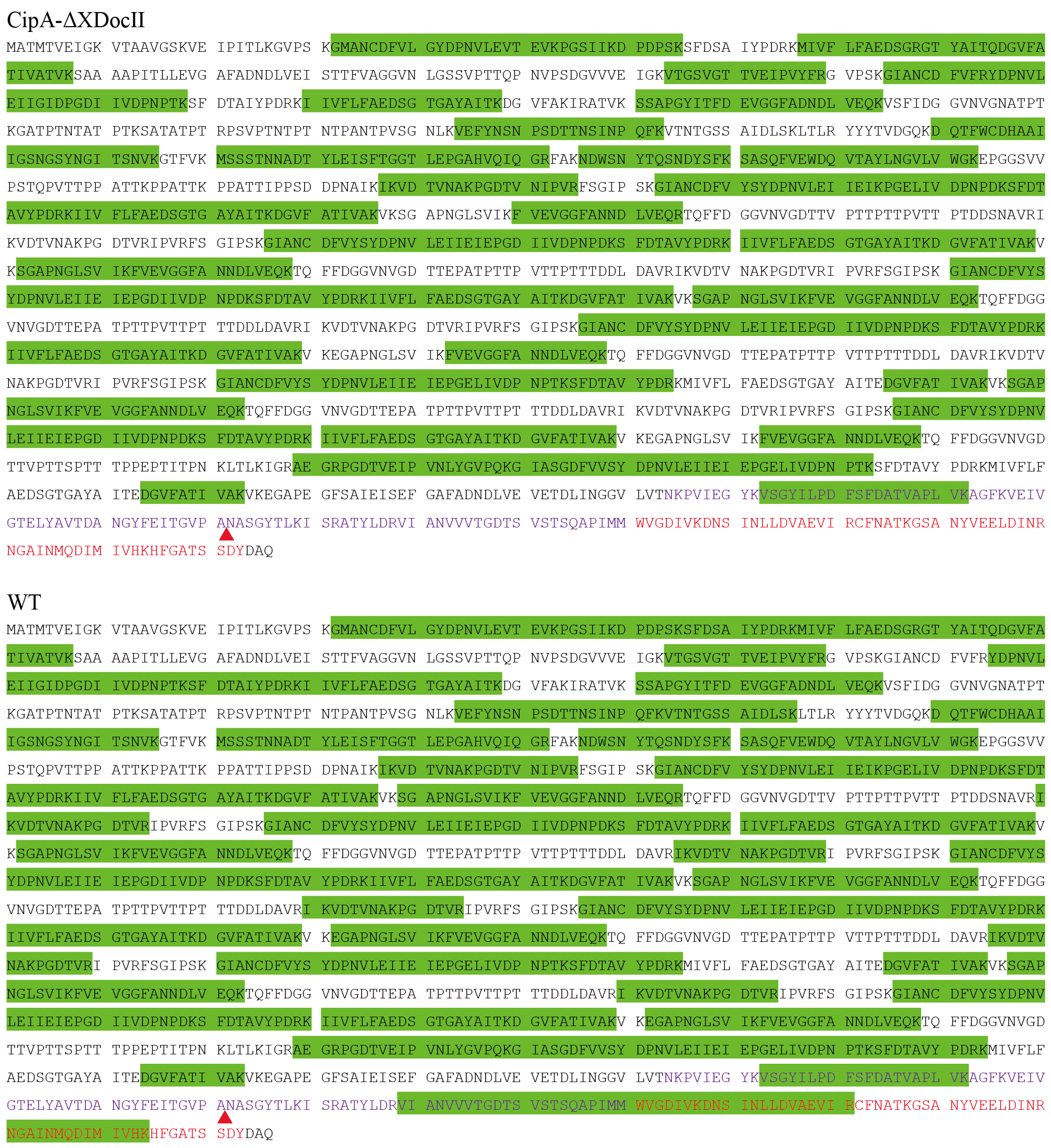


Additional file 7. Identified CipA peptides in the wild-type and CipA-ΔXDocII strains by mass spectroscopy analysis.

The green highlights indicate peptides of CipA detected by mass spectroscopy. The amino acid sequences shown in purple and red are predicted to be part of the X-module and DocII module, respectively. The insertion site of the CipA3740s targetron is indicated by a red triangle in the wild-type and CipA-ΔXDocII sequences. No peptides after the targetron insertion site are detected in CipA-ΔXDocII, indicating deletion of the XDocII module, whereas two peptides are found in the same region in wild-type.
