# Supplementary material for: The contribution of cellulosomal scaffoldins to cellulose hydrolysis by Clostridium thermocellum analyzed by using thermotargetrons
Source: Biotechnol Biofuels. 2014 May 29;7:80. doi: 10.1186/1754-6834-7-80 (PMC4045903; doi:10.1186/1754-6834-7-80)
Supplement: Additional file 9 — SDS-PAGE of supernatant proteins of wild-type and mutant strains. [file 1754-6834-7-80-S9.docx]

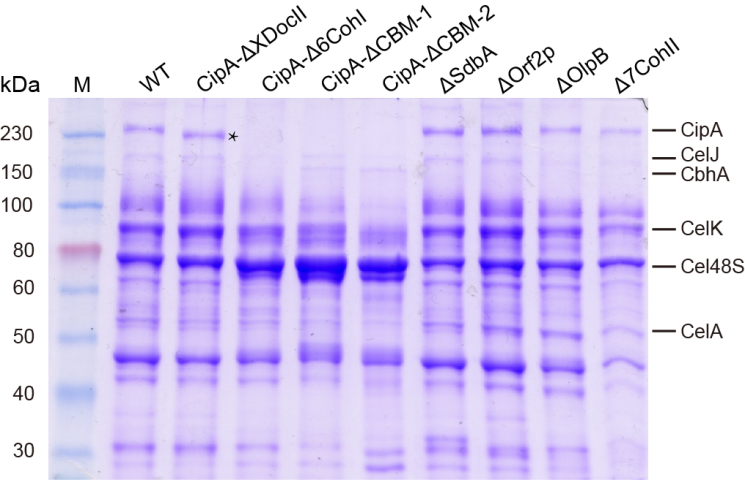


## Additional file 9. SDS-PAGE of supernatant proteins of WT and mutant strains.

Extracellular proteins of strains listed at the top of the gel were concentrated from broth supernatants, and show similar compositions to cellulosomal proteins (see Figure 3). The band in CipA-ΔXDocII indicated by an asterisk was slightly smaller than wild-type CipA, and was identified by mass spectroscopy as a truncated CipA lacking an XDocII module. M, protein markers.
