# Supplementary material for: The contribution of cellulosomal scaffoldins to cellulose hydrolysis by Clostridium thermocellum analyzed by using thermotargetrons
Source: Biotechnol Biofuels. 2014 May 29;7:80. doi: 10.1186/1754-6834-7-80 (PMC4045903; doi:10.1186/1754-6834-7-80)
Supplement: Additional file 8 — Growth analysis of CipA-truncated and secondary scaffoldin-disrupted mutants with cellobiose as the carbon source. [file 1754-6834-7-80-S8.docx]

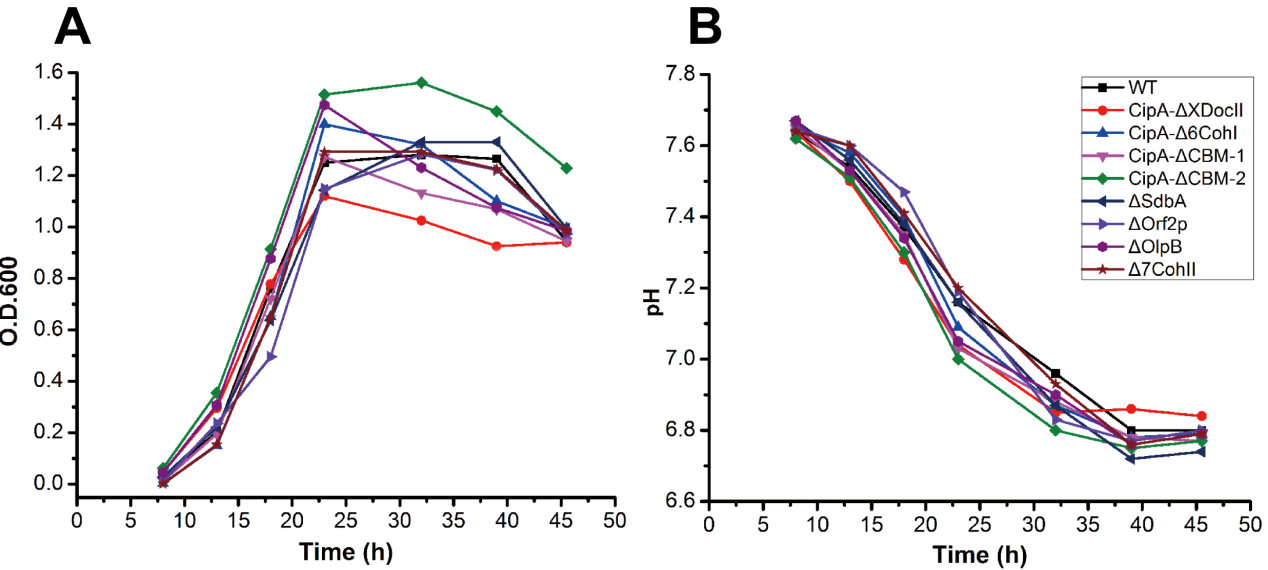


## Additional file 8. Growth analysis of CipA-truncated and secondary scaffoldin disrupted mutants with cellobiose as carbon source.

(A) Cell growth of *C. thermocellum* strains was measured by optical density at 600 nm. (B) The pH of the culture medium for all strains varied with culture time in a similar way. Two independent replicates were used to calculate the mean values.
