# Supplementary material for: The contribution of cellulosomal scaffoldins to cellulose hydrolysis by Clostridium thermocellum analyzed by using thermotargetrons
Source: Biotechnol Biofuels. 2014 May 29;7:80. doi: 10.1186/1754-6834-7-80 (PMC4045903; doi:10.1186/1754-6834-7-80)
Supplement: Additional file 11 — Quantification of polycellulosomal protuberances on the cell surfaces of wild-type C. thermocellum and secondary scaffoldin mutants. [file 1754-6834-7-80-S11.docx]

## Additional file 11. Quantification of polycellulosomal protuberances on the cell surfaces of wild-type *C. thermocellum* and secondary scaffoldin mutants.

The mean number of cellulosomes per μm^2^ was determined by counting the number of cellulosomes in three different 0.1 μm x 0.1 μm cell surface areas of each strain and multipling the number by 100. The error bars indicate the standard deviations.
