# Supplementary material for: The contribution of cellulosomal scaffoldins to cellulose hydrolysis by Clostridium thermocellum analyzed by using thermotargetrons
Source: Biotechnol Biofuels. 2014 May 29;7:80. doi: 10.1186/1754-6834-7-80 (PMC4045903; doi:10.1186/1754-6834-7-80)
Supplement: Additional file 10 — Curve-fitting parameters used to calculate the Avicel consumption data. [file 1754-6834-7-80-S10.docx]

Additional file 10. Curve fitting parameters used to calculate the Avicel consumption data.

| Strains | Parameters used to fit the signoidal curve^*^ | | | | | Adjusted R-square |
| --- | --- | --- | --- | --- | --- | --- |
|  | *A_0_* | *A_t_* | *sl* | *ap* | *t_0_* |  |
| WT (DSM1313)  CipA-ΔXDocII  CipA-Δ6CohI  CipA-ΔCBM-1  CipA-ΔCBM-2  ΔSdbA  ΔOrf2p  ΔOlpB  Δ7CohII | 2.04  2.60  2.54  11.56  12.00  3.73  5.08  5.88  2.31 | 58.24  59.33  58.83  59.47  58.47  59.41  59.79  60.52  57.97 | -2.07  -3.63  -5.94  -22.66  -38.54  -2.12  -3.63  -6.81  -4.29 | 0.17  0.36  0.35  0.30  0.47  0.14  0.32  1.37  0.37 | 22.22  37.28  74.81  87.56  176.06  21.99  23.75  42.10  31.94 | 0.997  0.999  0.999  0.995  0.997  0.999  0.999  0.998  0.999 |

^*^ The sigmoidal curves are fitted based on a 5-parameter Richards equation $y\left(t\right)=A_{0}+\frac{A_{t}-A_{0}}{(1+e^{\frac{t_{0}-t}{sl}})^{ap}}$[1], in which *A_0_* is the lower horizontal value, *A_t_*, is the higher horizontal value, *t_0_* is the inflection point, *sl* is the slope at *t_0_*, and *ap* is the asymmetry parameter. The variable *t* is the cultivation time [2].

1. Richards FJ: **A flexible growth function for empirical use.** *J Exp Bot* 1959, **10:**290-300.

2. Olson DG, Giannone RJ, Hettich RL, Lynd LR: **Role of the CipA scaffoldin protein in cellulose solubilization, as determined by targeted gene deletion and complementation in *Clostridium thermocellum*.** *J Bacteriol* 2013, **195:**733-739.
